# Identification of new fucosyltransferase-8 inhibitors: An integrated biochemical, biophysical, and computational approach

**DOI:** 10.1371/journal.pone.0349302

**Published:** 2026-10-05

**Authors:** Muhammad Awais, Noor Rahman, Sumaira Javaid, Atia-tul- Wahab, Thirugnanasambandam Rajendran, M. Iqbal Choudhary, Humaira Zafar

**Affiliations:** 1 Dr. Panjwani Center for Molecular Medicine and Drug Research, International Center of Chemical and Biological Sciences, University of Karachi, Karachi, Pakistan; 2 H.E.J. Research Institute of Chemistry, International Center for Chemical and Biological Sciences, University of Karachi, Karachi, Pakistan; 3 Institute of Molecular Biology and Biochemistry, The University of Lahore, Lahore, Pakistan; 4 National Facility for Coastal & Marine Research (NFCMR) & Centre for Ocean Research (COR), Sathyabama Institute of Science and Technology, Chennai, Tamil Nadu, India; 5 Institute of Drug Discovery Technology, Ningbo University, Ningbo, Zhejiang, China; International Center For Chemical and Biological Sciences, PAKISTAN

## Abstract

The *α*-1,6-fucosyltransferase (FUT8) mediated core fucosylation is responsible for several pathophysiological processes, including prostate cancer progression. FUT8 is a promising therapeutic target owing to its effects on cell migration and its capacity to convert androgen-dependent prostate cancer cells into androgen-independent cells. This study, therefore, investigates potential FUT8 inhibitors as therapeutic options for prostate cancer by targeting FUT8-mediated core fucosylation using *in vitro*, biophysical, and computational approaches. Eight of the 265 compounds showed significant cytotoxicity against the prostate cancer (PC3) cell line in *in vitro* screening. These compounds demonstrated dose-dependent reductions in cell viability, with IC_50_ values ranging from 2.94 ± 0.04 to 75.82 ± 2.10 µM. These compounds were non-cytotoxic against the normal human fibroblast (BJ) cell line. Biophysical and *in silico* analyses were conducted on active compounds to obtain atomic-level structural details of interactions between FUT8 and ligands. Among these, compounds **1**, **3**, **6**, and **7** showed significant Saturation Transfer Difference Nuclear Magnetic Resonance (STD-NMR) effects and induced a decrease in the melting temperature (Tm) of FUT8 in the differential scanning fluorimetry (DSF) assay (0.25°C–9°C), indicating ligand-associated destabilization of the protein. Molecular docking and molecular dynamics simulation results showed that compounds **1**, **2**, **3**, **4**, **6**, and **7** stably interacted with the FUT8 binding site. These compounds also exhibited anti-migratory activity in a scratch cell assay, as indicated by scratch closure of only 3.14% to 14.18%, compared with 45.45% to 100% in the control group. Our study identified compounds **1**, **3**, **6**, and **7** as promising leads against the FUT8 enzyme. Further mechanistic and *in vivo* studies are required to evaluate their mechanisms of action for FUT8-driven prostate cancer progression.

## Introduction

Fucosyltransferases (FUTs) are *bi*-substrate enzymes responsible for the transfer of the fucose moiety from GDP-fucose (donor substrate) to the *N*- and *O*-linked glycans (acceptor substrate). This is termed as fucosylation and is the terminal step in the production of glycoconjugates. To date, 13 FUTs have been identified that differ in their acceptor substrates, including FUTs 1−11 (transfer to *N*-glycan), and 2 POFUT 1,2 (transferred to *O*-glycan). Among FUTs, FUT1-FUT2 and FUT3-FUT7 catalyze the formation of α-1,2 and α-1,3 linkages, respectively. FUT8 is the unique member solely responsible for core fucosylation (*α*-1,6 linkage) of proteins, a process that involves the addition of a fucose sugar to the innermost component of *N*-linked glycans [1].

Previous research highlights the role of core fucosylation in regulating both intracellular and extracellular protein trafficking and function [2,3]. FUT8 has been implicated in various biological and tumor characteristics and is found to be upregulated in several cancers, including lung [4], liver [5], colorectal [6], ovarian [7], breast [8], melanoma [9], thyroid [10], pancreatic [11], and prostate [12]. Core fucosylation, caused by FUT8, is also known for modulating the humoral immune response by altering antibody-dependent cellular cytotoxicity [13]. The upregulation of FUT8 across various cancer types suggests that inhibiting its activity could be a promising approach to enhance antitumor immune responses [14].

Previous studies have shed light on the association between aberrant fucosylation and aggressive prostate cancer, highlighting the therapeutic potential of FUT8 in this context [15–19]. Utilizing prostate cancer models, researchers have demonstrated that the overexpression of FUT8 was sufficient to transform androgen-dependent LAPC4 prostate cancer cells into androgen-resistant cells [18] while significantly enhancing their migratory abilities. On the other hand, knocking down the *FUT8* gene in PC3 cells, an androgen-independent prostate cancer cell line, resulted in a significant reduction in the migratory capacity of PC3 cells [19].

To date, no clinical fucosyltransferase inhibitor has been reported, despite the use of various strategies. One approach involves mimicking the natural substrate guanosine diphosphate fucose (GDP-Fuc), while alternative methods focus on creating fucose derivatives that are metabolized through the salvage pathway, targeting fucosyltransferases *via* competitive inhibition [20]. One such inhibitor, 2-fluoro fucose (SGN-2FF), is metabolized *via* the salvage pathway and has exhibited promising anti-cancer effects in both *in vitro* and *in vivo* studies [20]. SGN-2FF is a small-molecule inhibitor of fucosylation that directly and indirectly affects immune cells, tumor cells, and the tumor microenvironment [21]. The inhibitor is passively transported across the cell membrane, deprotected by esterases, and metabolized to its corresponding GDP analog [22]. SGN-2FF has been employed in various mouse models as an orally bioavailable inhibitor and has undergone testing in a phase 1 multicenter clinical trial for patients with advanced solid tumors (NCT# 02952989). However, the study was terminated after three years due to the overall benefit/risk profile, with no further details disclosed. Therefore, there is an unmet need to identify new fucosyltransferase inhibitors that are more efficacious or offer a more favorable benefit-to-risk ratio.

Therefore, this study aimed to identify potential inhibitors of FUT8 through an integrated methodology combining *in vitro*, biophysical, and computational techniques. These include screening structurally diverse compounds (such as flavonoids, alkaloids, and nucleoside analogs, among others) from our in-house library for anti-proliferative activity against the PC3 cell line. Interactions between the hit compounds and the FUT8 enzyme were explored using Saturation Transfer Difference Nuclear Magnetic Resonance (STD-NMR), differential scanning fluorimetry (DSF), and computational studies. This was followed by assessing the anti-migratory potential of these compounds *via the* scratch cell assay. This integrative approach addresses the failure of GDP-Fuc mimetics and 2FF by combining biochemical assays, DSF, STD-NMR, and molecular dynamics to identify novel scaffolds. The hit compounds identified through this systematic approach substantially contribute to the development of novel therapeutic options for prostate cancer by targeting FUT8-mediated core fucosylation.

## Materials and methods

### Materials

The human prostate cancer (PC3; CRL-1435^™^, ATCC) and fibroblast (BJ; CRL-2522^™^, ATCC) cell lines were obtained from the BioBank, and the compounds were obtained from the Drug Bank of Dr. Panjwani Center for Molecular Medicine and Drug Research, ICCBS. Roswell Park Memorial Institute (RPMI)-1640 (Cat# 11875093), penicillin-streptomycin (Cat# 15070-063), Trypan Blue Stain (0.4%; Cat# 15250-061), and heat-inactivated fetal bovine serum (FBS; Cat# 10500-064) were purchased from Gibco, USA. Human recombinant FUT8 protein (Cat# 11326-H08B) was purchased from SinoBiological, USA. DMSO (HPLC grade; Cat# 34869-2.5L) and guanosine 5′-diphosphate-*β*-L-fucose sodium salt (Cat# G4401) were purchased from Sigma-Aldrich, USA. Tris (*d*_*11*_, 98%; Cat# DLM-1814-5) was procured from Cambridge Isotope Laboratories, Inc., USA. Deuterium oxide (D_2_O; Cat# 1069-1X100ML) and dimethylsulfoxide (DMSO-*d*_*6*_; Cat# 1088-1X100ML) were purchased from ARMAR Isotopes, Switzerland. Fluorescent dye Sypro®Orange (Cat# S6650) was obtained from Invitrogen Life Technology, USA.

### Compound library

A total of 265 fully characterized compounds were obtained from the *in-house* library of the Drug Bank of Dr. Panjwani Center for Molecular Medicine and Drug Research. These compounds were screened at a concentration of 100 μM, prepared in DMSO.

### Cell culture

The PC3 [23] and BJ [24] cell lines were cultured in RPMI-1640 and DMEM, with 10% FBS and 1% penicillin/streptomycin in a 5% CO_2_ incubator at 37°C. Trypsin-EDTA (0.05%) was used to routinely passage the cells at a 70–80% confluency in T75 flasks. All the assays were performed with exponentially growing cells.

### Cell proliferation assay

Compounds were screened for their anti-cancer and anti-cytotoxic ability using MTT (dimethylthiazol-2-yl)-2,5-diphenyltetrazolium bromide) assay [25]. Exponentially growing PC3 and BJ cells were plated at a density of 7,000 cells per well in a 96-well plate and incubated to form a monolayer. After a 24-hour incubation, the cells were treated with compounds at a concentration of 100 µM. Following a 48-hour treatment period, the MTT dye (0.5 mg/mL) [26,27] was added to each well, followed by 4 hours of incubation. The resulting formazan crystals were then dissolved in DMSO for spectrophotometric analysis at 550 nm. Controls included a standard vehicle dilution (1% DMSO) and empty wells for baseline readings. The independent experiments were performed (biological replicates, n = 3; technical replicates, n = 3), and the percent inhibition was determined using the formula:


$$\begin{array}{c} Percent\;inhibition\;(\%)\;=\text{1}\text{0}\text{0}-\left\{\left(\frac{Absorbance\;of\;treated\;cells-Absorbance\;of\;negative\;control}{Absorbance\;of\;positive\;control-Absorbanceof\;negative\;control}\right)\times \text{1}\text{0}\text{0}\right\} \end{array}$$

### Differential Scanning Fluorimetry (DSF)

Differential scanning fluorimetry (DSF) studies were conducted using a CFX96 real-time PCR system, in accordance with the protocol reported previously [28,29]. In summary, samples comprising protein (1 μM), ligand (1 mM), and Sypro®Orange (1:1000) were added to a PCR tube and subjected to heating at a rate of 0.3°C/min, ranging from 20 to 99.9 °C. All reagents were dissolved in Tris buffer (20 mM Tris-HCl, pH 8.0). The fluorescence of the dye was monitored utilizing the Hex channel as a function of temperature. The resulting data were processed and analyzed using CFX Manager™. Three independent experiments were performed for each compound.

### STD-NMR experiment

STD-NMR experiments were conducted on a 600 MHz Bruker Neo Avance spectrometer. The magnet was equipped with an inverse triple-resonance cryoprobe and automated sample changer (SampleCase™, Bruker, Switzerland). Compounds were prepared in DMSO-*d*_*6*_, and all experiments were conducted using a 20 mM deuterated *Tris-*_*d11*_ buffer (pH 8.0) at 298K. The procedure involved initial ^1^H-NMR and STD-NMR experiments of the ligand (1 mM) in 550 µL of buffer (to prevent false positives), followed by the addition of the FUT8 protein (1 µM) and subsequent STD-NMR assessments.

The standard pulse program *stddiffesgp.3* (Bruker Biospin, Switzerland) with water signal suppression by excitation sculpting was used for STD-NMR experiments. Key experimental parameters included a 3.1 relaxation delay, a 30 ms spin-lock pulse, a 3.0 s saturation duration, and −0.50 ppm *on-resonance* and 35 ppm *off-resonance* offsets. The difference spectrum was obtained by subtracting the *on-resonance* spectrum from the *off-resonance* spectrum. The difference spectrum showed signals for the ligand protons that interact with the protein [27,30]. To quantify these interactions, the STD amplification factor for each proton was determined using the formula [27,31]:


$$\begin{array}{c} STD\;Amplification\;Factor=\left(\frac{Istd}{Io}\right)\;\times Ligand\;Excess, \end{array}$$

where Io and Istd represent the signal strengths in the *off-resonance* and difference spectra, respectively.

### Computational docking and simulations

#### Receptor and ligand preparation.

The structure of human FUT8, co-crystallized with GDP (PDB ID: 6TKV), was retrieved from the Protein Data Bank (https://www.rcsb.org/). The compound structures were prepared, and their 3D coordinates were generated using the LigPrep module of the Schrödinger software suite (version 2022−1). Ligand preparation involved energy minimization, chirality correction, and addressing tautomeric and ionization states, generating 32 conformations per ligand. Protein preparation involved refining the FUT8 structure through preprocessing steps, including removing non-essential molecules, adding missing atoms, and adjusting hydrogens. This was followed by optimization to refine hydrogen bonds and correct protonation states, and minimization using the OPLS4 force field. A receptor grid was then generated around the co-crystallized GDP, ensuring comprehensive coverage and precise site representation, which was validated through careful inspection. This meticulous preparation facilitated subsequent computational docking studies and analyses.

#### Molecular docking.

Molecular docking of the prepared compound library with human FUT8, co-crystallized with GDP (PDB ID: 6TKV), was performed utilizing the Glide module of the Schrödinger software suite with extra precision (XP) [27,32] for enhanced accuracy. The docking process began with a comprehensive conformational analysis of each ligand within the receptor’s active site, followed by the application of multiple scoring functions (including Coulombic, van der Waals, and hydrogen-bonding evaluations) to ascertain ligand binding affinities. All XP docking parameters were set to default values, and post-docking minimization was performed to refine ligand geometries and optimize receptor-ligand interactions for stability analysis. Docking was validated by re-docking GDP. RMSD 0.152 Å confirmed the accuracy of the docking protocol (Fig S1 in S1 File). Post-docking analysis of ligand-protein interactions was conducted to check the stability of the complexes.

#### Energy calculation.

The binding energies for the docked complexes involving the human FUT8 and the respective ligands were evaluated using the Molecular Mechanics Generalized Born Surface Area (MM-GBSA) [33] in the Prime [34] module of the Schrödinger software suite. The calculation was carried out using the OPLS3e force field [35] with the variable-dielectric generalized Born (VSGB 2.1) solvation model, incorporating empirical corrections to model the directionality of solvent effects. All parameters were set to default values to ensure consistency with widely accepted practices.

#### Molecular dynamics simulation.

The docked ligand-protein complexes were further analyzed through molecular dynamics (MD) simulations [36,37] using the Desmond [37,38] module of the Schrödinger software suite [37]. Simulation systems were set up in orthorhombic boxes, with a 10.0 Å buffer in all dimensions, using the SPC water model to solvate the complexes. Charge equilibrium within the system was maintained by adding Na^+^/Cl^-^ ions, supplemented with an additional saline concentration of 0.15 M NaCl. The system was equilibrated using the NPT ensemble, maintaining a temperature of 310K and a pressure of 1 Bar. The OPLS4 force field [39] was used throughout the simulation, with integration steps proceeding in 2.0-femtosecond increments, facilitating the precise analysis of the complex’s behavior. Subsequent trajectory data from the MD simulations were systematically analyzed to evaluate the complex’s flexibility, structural stability, and key interactions.

### Scratch cell assay

The cellular migration evaluation was conducted using a scratch cell assay [40]. PC3 cells, revived in RPMI-1640 with 10% FBS, were seeded into six-well plates at approximately 3 × 10^5^ cells per well and incubated overnight. Subsequently, a 200 μL pipette tip was used to create a scratch across the central region of each well. Post-scratch, the cells were washed twice with PBS and treated with the respective compounds at their IC_50_ concentration. Observation checkpoints were established at the onset, 24, and 48 hours, during which images of the designated areas were captured with a phase contrast microscope at 10X magnification. Scratches of treated cells were compared with those of the non-treated control cells. The experiments were performed in triplicate (biological replicates, n = 3). Image analysis was facilitated through ImageJ software (NIH, USA), focusing on quantifying the percent scratch closure by applying the following formula:


$$\begin{array}{c} \text{Percent closure of scratch}=\frac{\text{Mean area at }\text{0}\text{ h}-\text{Mean area at test h}}{\text{Mean area at }\text{0}\text{ h}}\times \text{100} \end{array}$$

### Statistical analysis

For the anti-proliferative activity, GraphPad Prism 8.0 (Dotmatics, USA) was used for statistical analysis. After normalizing the data, non-linear regression was used, and the results were presented as the mean IC_50_ ± standard error of the mean (SEM). A one-way ANOVA, followed by Dunnett’s test, was used for group comparison, and differences were deemed statistically significant at *p* < 0.05. To process the NMR data, 4.3.1 (Bruker, Biospin GmbH, Germany) was used. For the thermal shift assay, the fluorescence of the Syprorange dye as a function of temperature was recorded using the HEX (Ex/Em: 538/555 nm) channel, and the data were analyzed using CFX 96 Manager^TM^ software (Bio-Rad Laboratories, USA). The magnitude of thermal shift was determined in terms of melting temperature (Tm) of the protein, both in the absence and in the presence of the ligand.

## Results and discussion

### Anti-proliferative effects in prostate cancer (PC3) cell line

The PC3 cell line is an androgen-independent cancer cell line with high metastatic potential and high expression of FUT8 [19]. Therefore, this cell line was selected to analyze the anti-cancer effects of a library of commercially available compounds using an MTT assay. A total of 265 compounds were screened, among which eight compounds were found to be active, as presented in Fig 1. Compounds **1** and **2** showed potent anti-proliferative effects with IC_50_ values of 2.94 ± 0.04 and 3.07 ± 0.06 µM, respectively. Compounds **3** and **4** showed significant anti-proliferative effects with IC_50_ values of 19.42 ± 0.59 and 19.76 ± 0.61 µM, respectively. Compounds **5** and **6** showed moderate anti-proliferative effects with IC_50_ values of 41.33 ± 0.41 and 49.85 ± 1.23 µM, respectively. Furthermore, compounds **7** and **8** exhibited a moderate anti-proliferative effect with IC_50_ values of 64.21 ± 1.95 and 75.82 ± 2.10 µM, respectively (Fig S2 in S1 File). The activities of these compounds were compared with those of 2-fluorofucose (IC_50_: 259.70 ± 4.68 µM), used as a reference compound in the study, and all results were found to be statistically significant (*p* < 0.0001). IC_50_ ± SEM of all compounds was calculated from three independent experiments (n = 3), and their dose-dependent response curves are presented in supplementary Fig S3 in S1 File.

**Fig 1:**
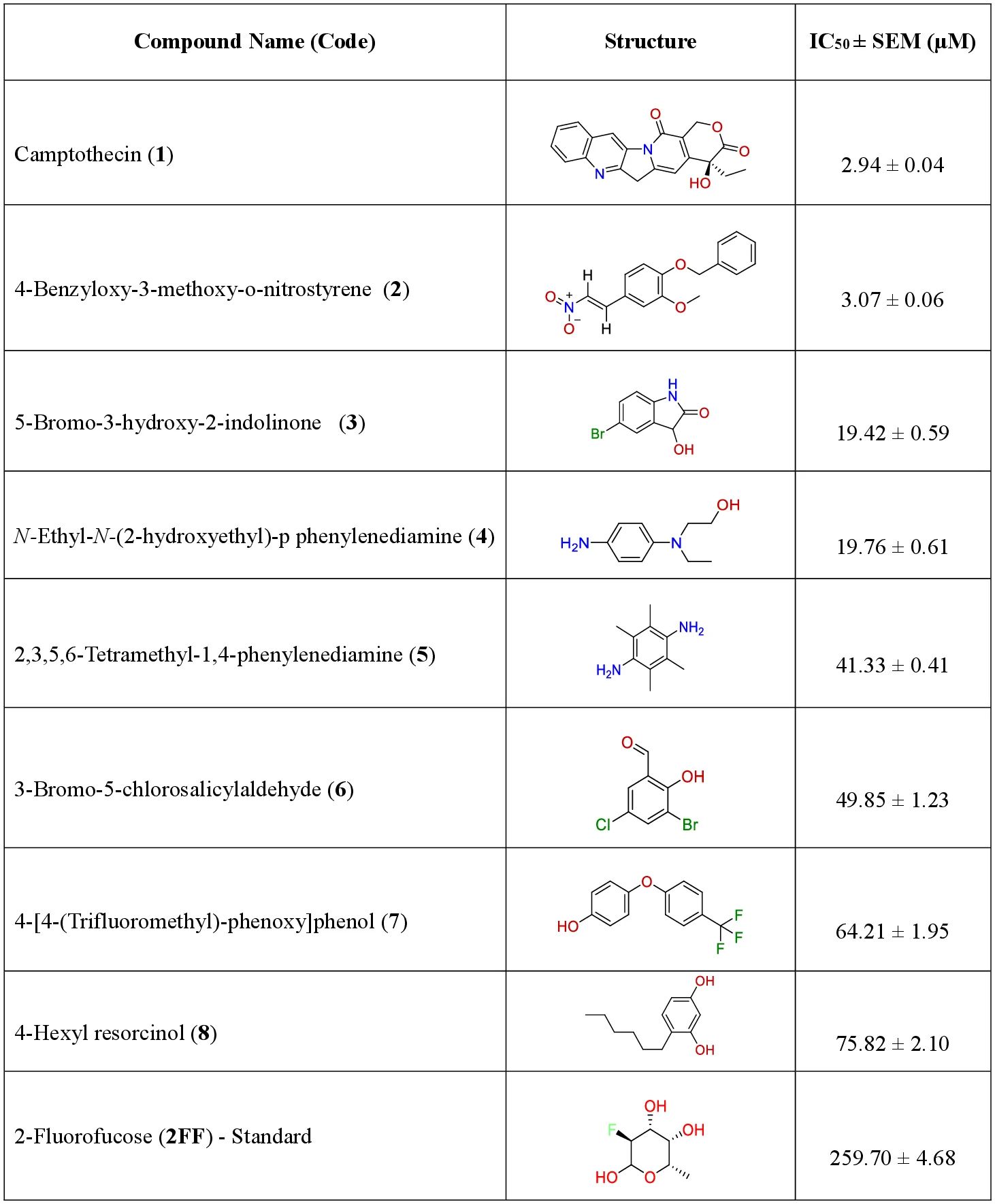
Names, structures, and the IC_50_ values of compounds active against the PC3 cell line.

### Cytotoxicity effects in the human fibroblast (BJ) cell line

The cytotoxicity profile against the human fibroblast BJ cell line was also analyzed, and these compounds were found to be non-cytotoxic to normal cells.

### Ligand-receptor interactions elucidation using a multidisciplinary approach

Since the compounds were identified using the PC3 cell line, further exploration of the direct interactions between the identified anti-proliferative compounds and the recombinant FUT8 enzyme was conducted. Biophysical studies (DSF and STD-NMR) were performed to investigate these interactions. These studies were further supported by computational analyses, including molecular docking, MM-GBSA energy calculations, and molecular dynamics simulations.

### Analysis by Differential Scanning Fluorimetry (DSF)

Differential Scanning Fluorimetry (DSF) is a fluorescence-based thermal shift assay that assists in evaluating the stability of the protein [28,29]. It uses a hydrophobic fluorescent dye that binds to proteins as they unfold, and fluorescence changes are quantified as a function of the melting temperature (Tm). Changes in Tm upon the addition of ligand reflect the stability index of the protein. Ligand binding to a protein may result in stabilizing or destabilizing the tertiary fold of the protein, which is reflected by an increase or decrease in the Tm, respectively.

GDP, being an enzyme substrate, exhibited an increase in Tm from 46 to 49°C, indicating stabilization of the tertiary fold of the protein. However, 2FF and all of the identified compounds caused a decrease in the Tm (≥ 0.5°C) of the protein (Table S1 in S1 File). These observations suggest interaction of the compounds with FUT8 and indicaFte ligand-associated destabilization of its tertiary structure, with the largest thermal shift observed for compounds **7** and **8** (Fig 2). Compound **4** caused protein aggregation; therefore, no effect was observed in DSF.

**Fig 2:**
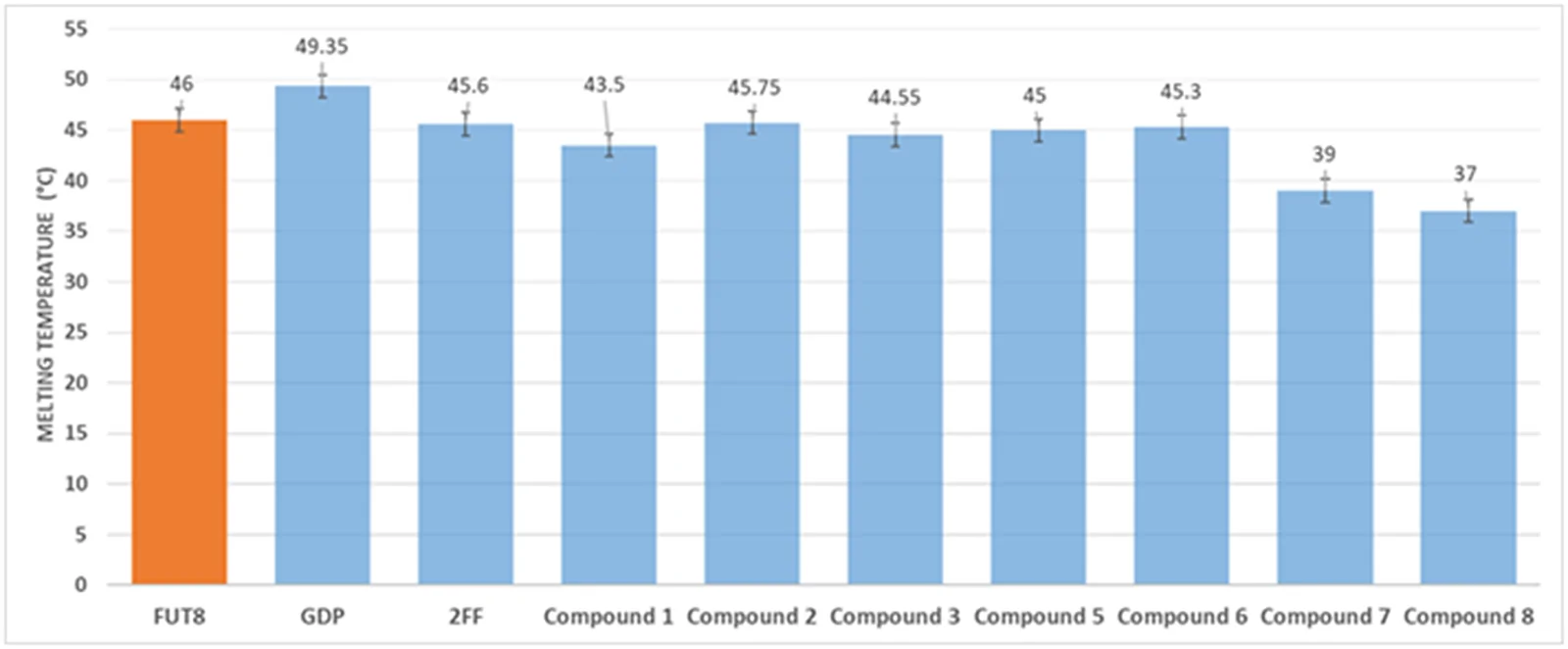
Graphical representation of Tm values of FUT8 upon addition of compounds. The Tm value for FUT8 alone is in orange. Blue bars correspond to compounds whose addition showed a change in Tm of FUT8.

### Ligand-receptor interactions elucidation by STD-NMR studies

In STD-NMR studies, the H-5′/H-5′′ of the substrate (GDP) demonstrated the highest STD amplification factor value and was used as a reference to achieve 100% saturation transfer (Table S2 in S1 File). The H-4′ of the ribose moiety received 83% relative saturation transfer, while the H-2′ and H-3′ with the same chemical environment received 49% relative saturation transfer. The group epitope mapping (GEM) analysis [27,29] indicated that the ribose was in close vicinity to the protein (Fig 3). The STD analysis of 2FF revealed that only the methyl group received saturation transfer, suggesting its close proximity to FUT8. The remaining protons (H-1/2/4/5) showed no STD effects (Fig 4).

**Fig 3:**
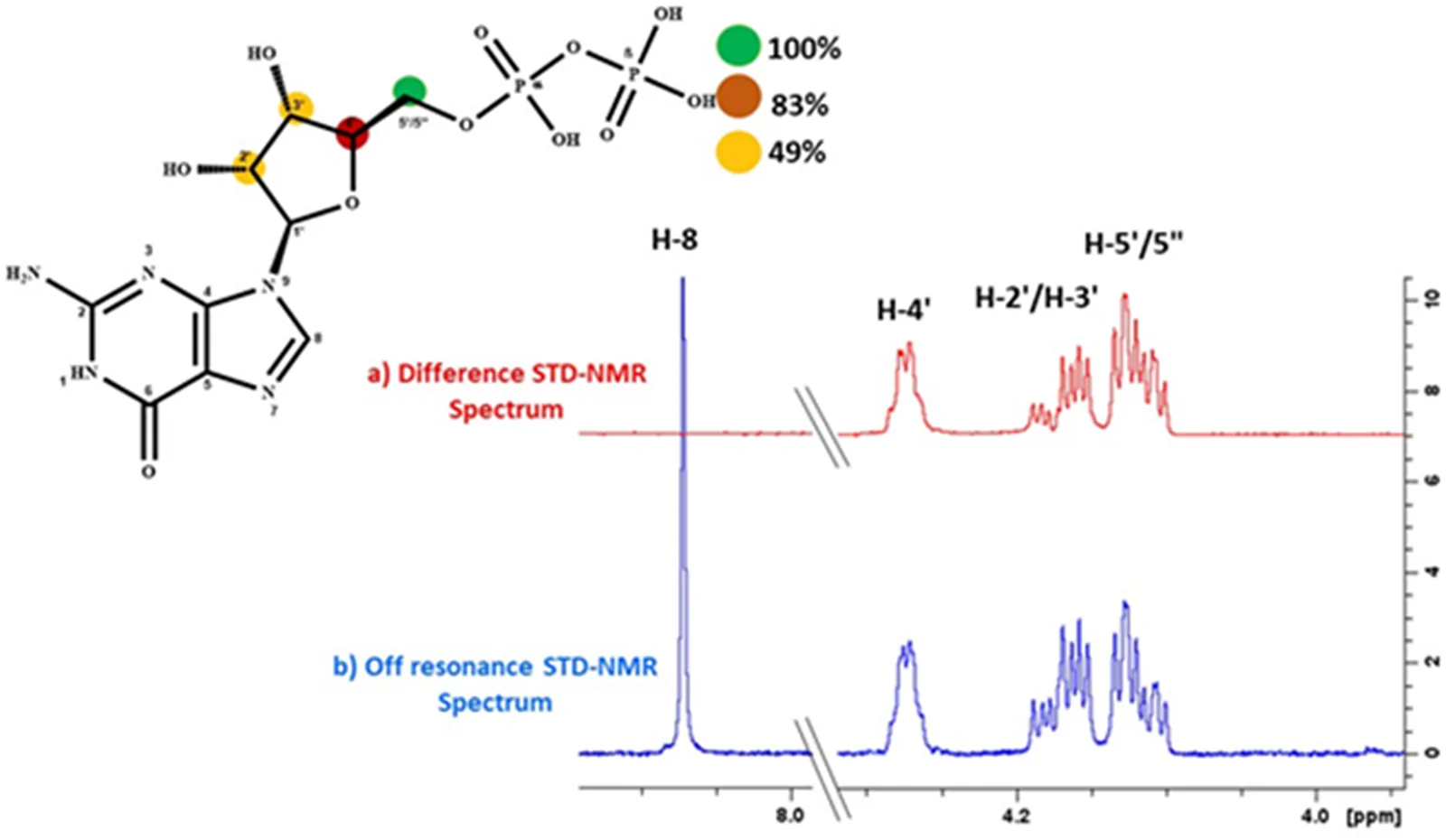
STD-NMR analysis of GDP with FUT8 protein: (a) is the off-resonance ^1^H-NMR spectrum of GDP, while (b) is the STD difference spectrum.

**Fig 4:**
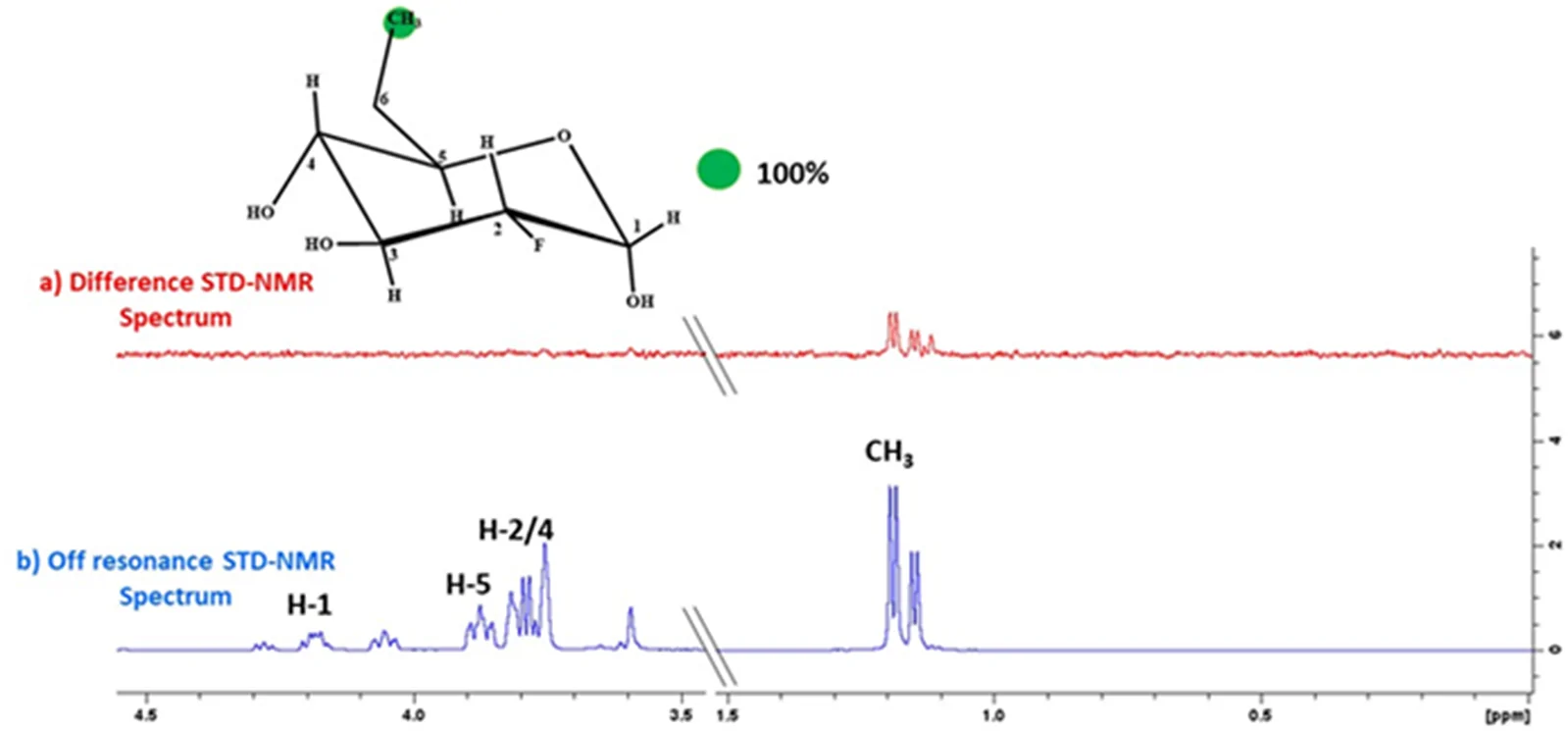
STD-NMR spectra of 2-Fluorofucose with FUT8 protein: (a) is the off-resonance ^1^H-NMR spectrum of 2-fluorofucose, while (b) is the STD difference spectrum.

Compound **1** has a pentacyclic quinoline structure and is considered a planar molecule. It consists of a pyrrolo[3,4-beta]-quinoline moiety conjugated to a pyridone moiety fused to a lactone ring. The H-11 displayed the highest STD amplification factor and was used as a reference to achieve 100% saturation transfer (Fig 5), followed by H-7 and H-9, which achieved 96% and 91% relative saturation transfer, respectively. The H-10 and H-12 showed relative saturation transfer of 75% and 52%, respectively. We were unable to assign a few protons due to their chemical shifts overlapping with those of the buffer and protein preservatives. The GEM analysis, based on available data, demonstrated that the quinoline moiety of compound **1** was in close proximity to the protein.

**Fig 5:**
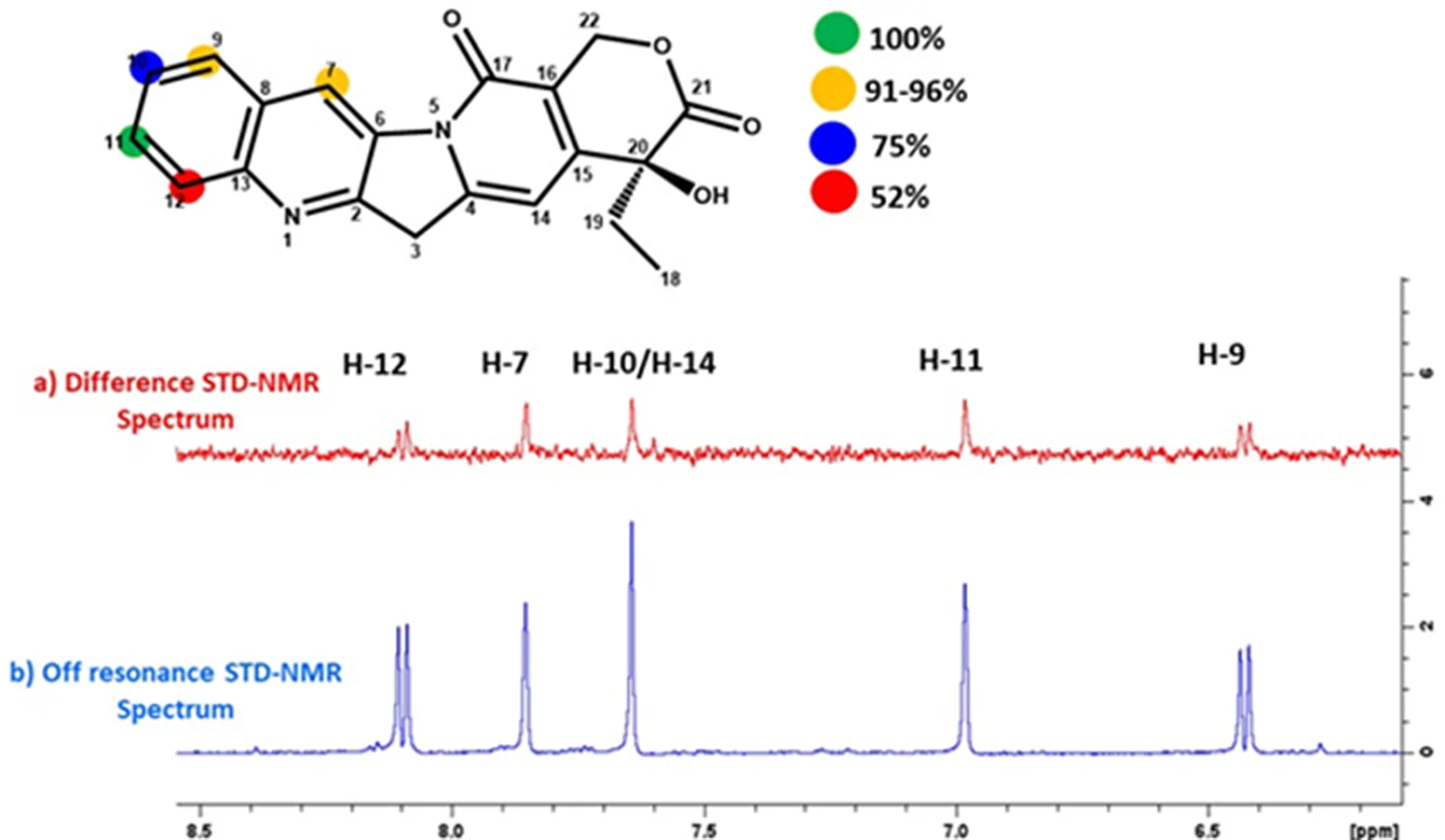
STD-NMR analysis of compound 1 with FUT8 protein: (a) is the off-resonance ^1^H-NMR spectrum of compound 1, while (b) is the STD difference spectrum.

Compound **3** is a small molecule and showed significant STD effects against FUT8. The H-4 with the highest STD integral was set to receive 100% saturation. This was followed by H-6 and H-7, which showed relative saturation transfers of 53% and 55%, respectively (Fig 6).

**Fig 6:**
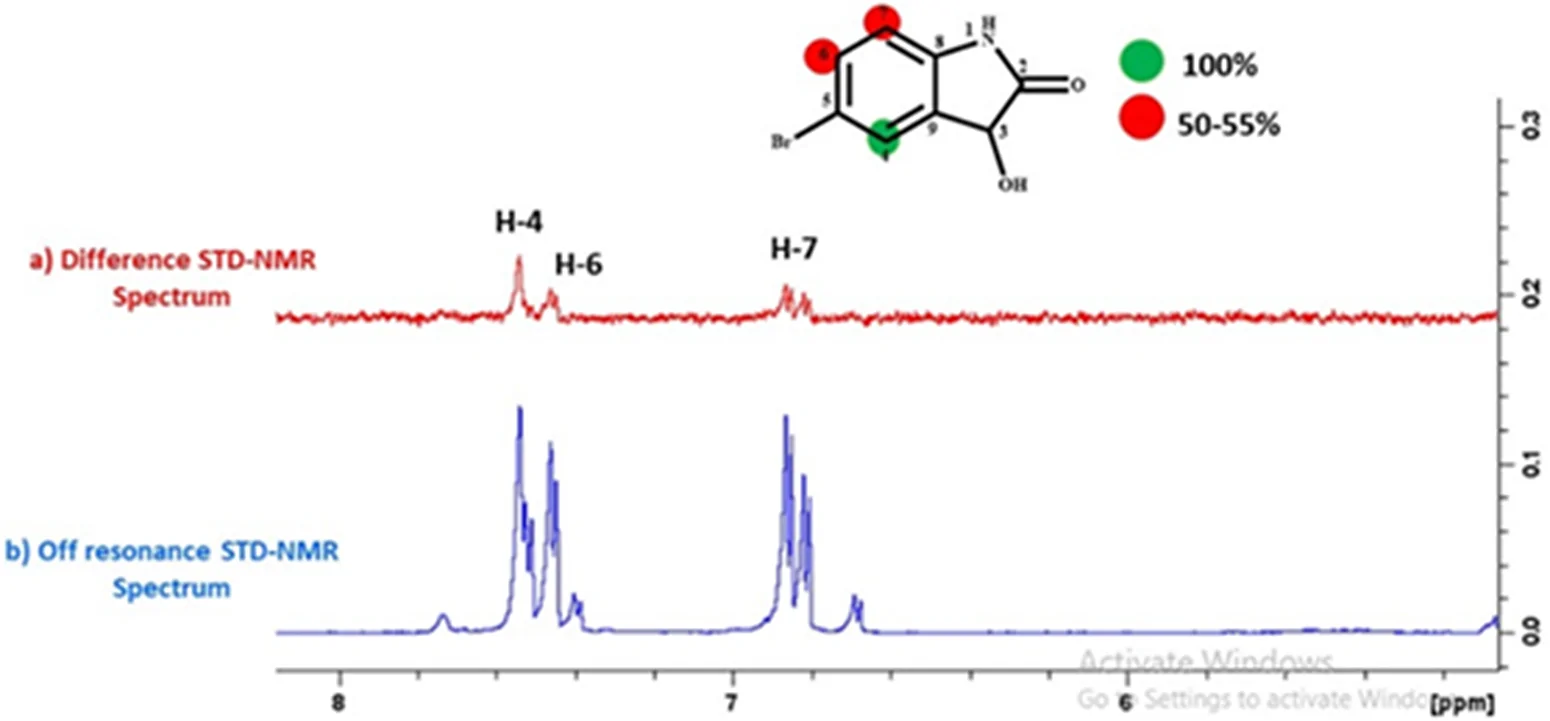
STD-NMR analysis of compound 3 with FUT8 protein: (a) is the off-resonance ^1^H-NMR spectrum of compound 3, while (b) is the STD difference spectrum.

Compound **5** is an aromatic ring substituted with four methyl groups and two amine groups. Given the symmetry of compound **5**, all the methyl groups received equivalent saturation transfer (100%) (Fig 7).

**Fig 7:**
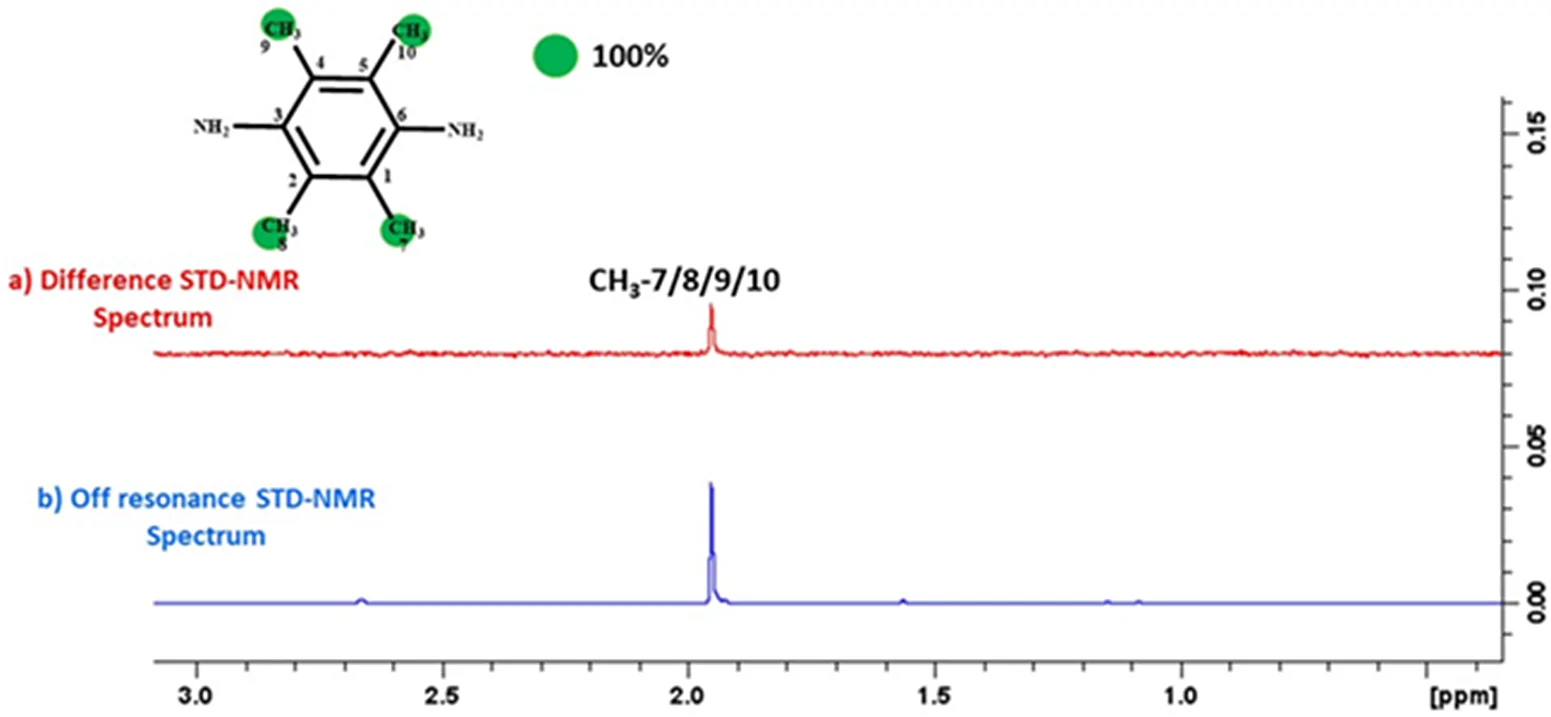
STD-NMR analysis of compound 5 with FUT8 protein: (a) is the off-resonance ^1^H-NMR spectrum of compound 5, while (b) is the STD difference spectrum.

Compound **6** is a relatively small molecule, and all its protons showed interactions in the STD spectrum. The aldehydic proton (H-1′) exhibited the highest intensity signal and was set to achieve 100% saturation from the protein, followed by H-6 and H-4, which received 54% and 34% relative saturation transfer, respectively (Fig 8).

**Fig 8:**
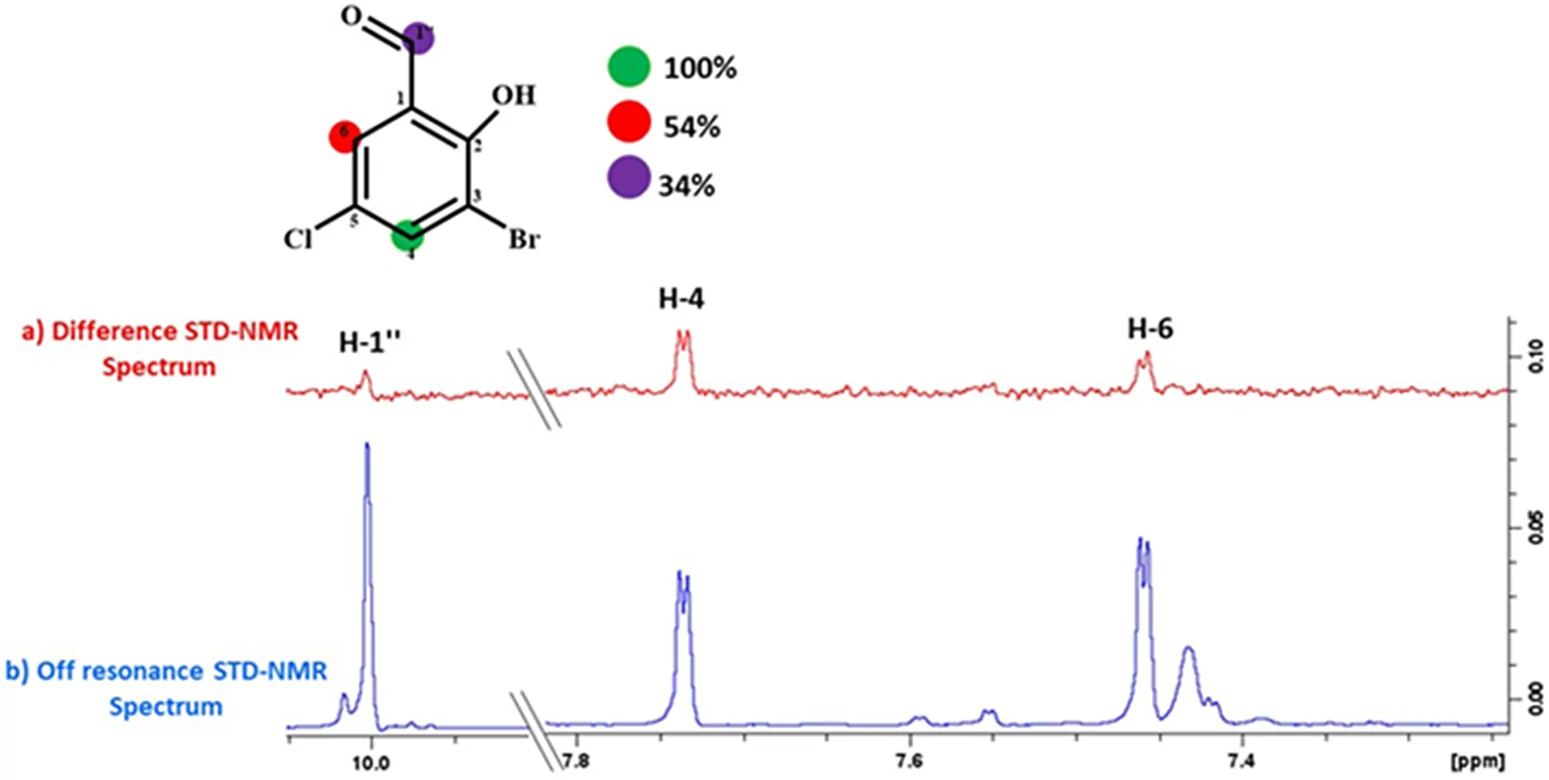
STD-NMR analysis of compound 6 with FUT8 protein: (a) is the off-resonance ^1^H-NMR spectrum of compound 6, while (b) is the STD difference spectrum.

Compound **7** showed substantial STD effects with the highest saturation (100%) received by H-2’/H-6’. This was followed by H-2/H-6, which exhibited a 97% relative saturation transfer. The rest of the protons, including H-3/H-5 and H-3′/H-5′, showed 93% and 95% relative saturation, respectively. Hence, all the protons of compound **7** indicated a significant STD effect (Fig 9)

**Fig 9:**
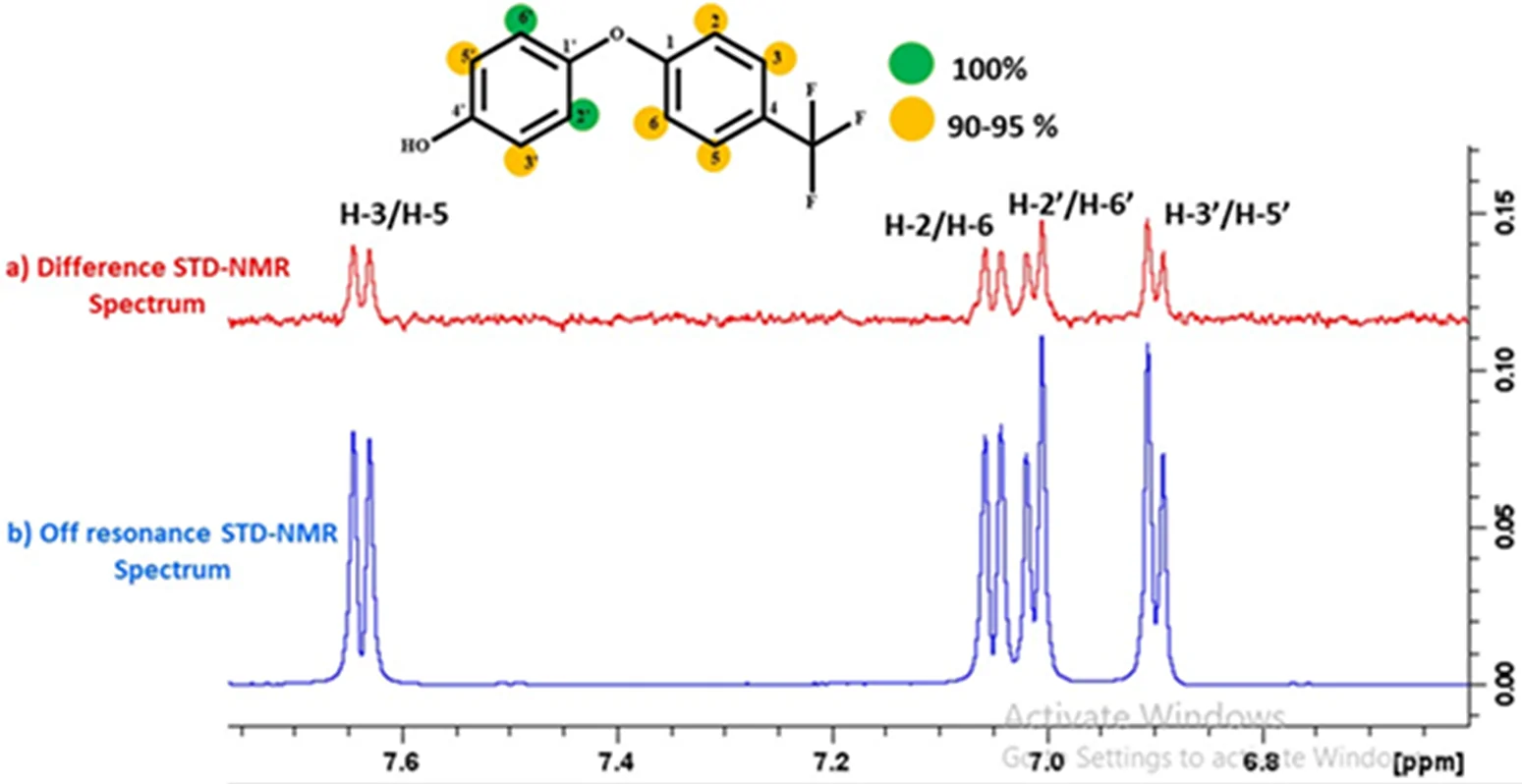
STD-NMR analysis of compound 7 with FUT8 protein: (a) is the off-resonance ^1^H-NMR spectrum of compound 7, while (b) is the STD difference spectrum.

The STD-NMR findings for compound **8** revealed a significant contribution of the aromatic ring to binding to the protein, with H-3/H-5 demonstrating 100% saturation transfer, highlighting the close proximity of the aromatic ring to the enzyme. This was followed by H-6′, which showed 39% relative saturation transfer, while the H-3′/H-4′/H-5′ received 24% relative saturation transfer (Fig 10).

**Fig 10:**
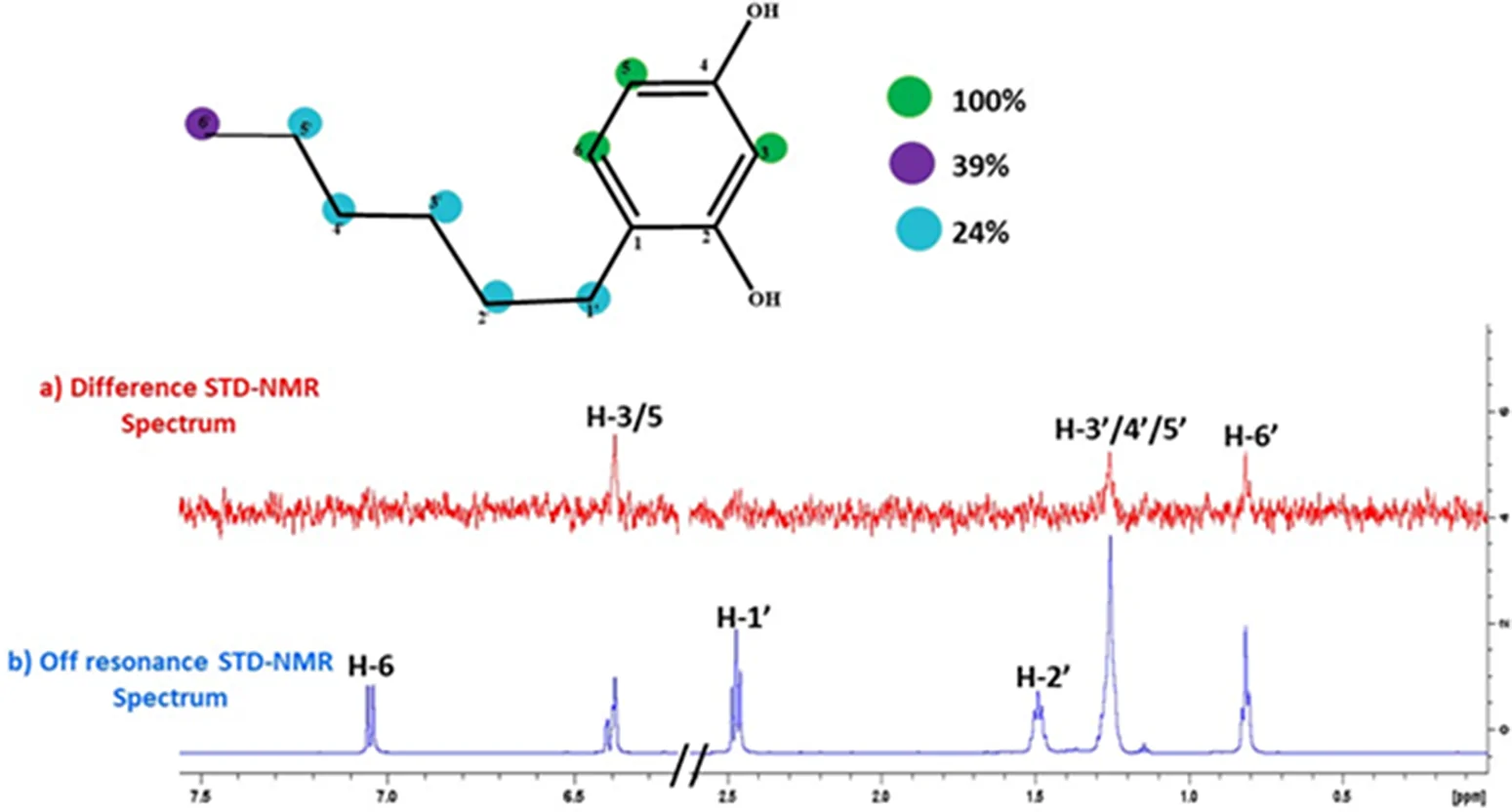
STD-NMR analysis of compound 8 with FUT8 protein: (a) is the off-resonance ^1^H-NMR spectrum of compound 8, while (b) is the STD difference spectrum.

Compounds **2** and **4** could not be studied *via* STD-NMR due to their limited solubility in the aqueous buffer.

### Computational studies

#### Guanosine-diphosphate (GDP).

GDP showed strong binding and stable complex formation, reflected by a docking score of −10.131 and a binding free energy of −64.49 kcal/mol. In the docking pose, the phosphate group of GDP formed hydrogen-bond interactions with Gly219, Arg365, Lys369, and Ser469, while the ribose moiety established hydrogen bonds with Tyr250. Moreover, the guanine base formed hydrogen bonds with His363 and Asp453 of FUT8, including an aromatic hydrogen bond with Asp453 (Fig 11A). A two-dimensional LigPlot interaction map showing hydrogen bonds together with distances is shown in Fig 11B. The docking scores, binding free energies, and hydrogen-bonding residues, along with their bond lengths for all the ligands, are given in Table S3 in S1 File.

**Fig 11:**
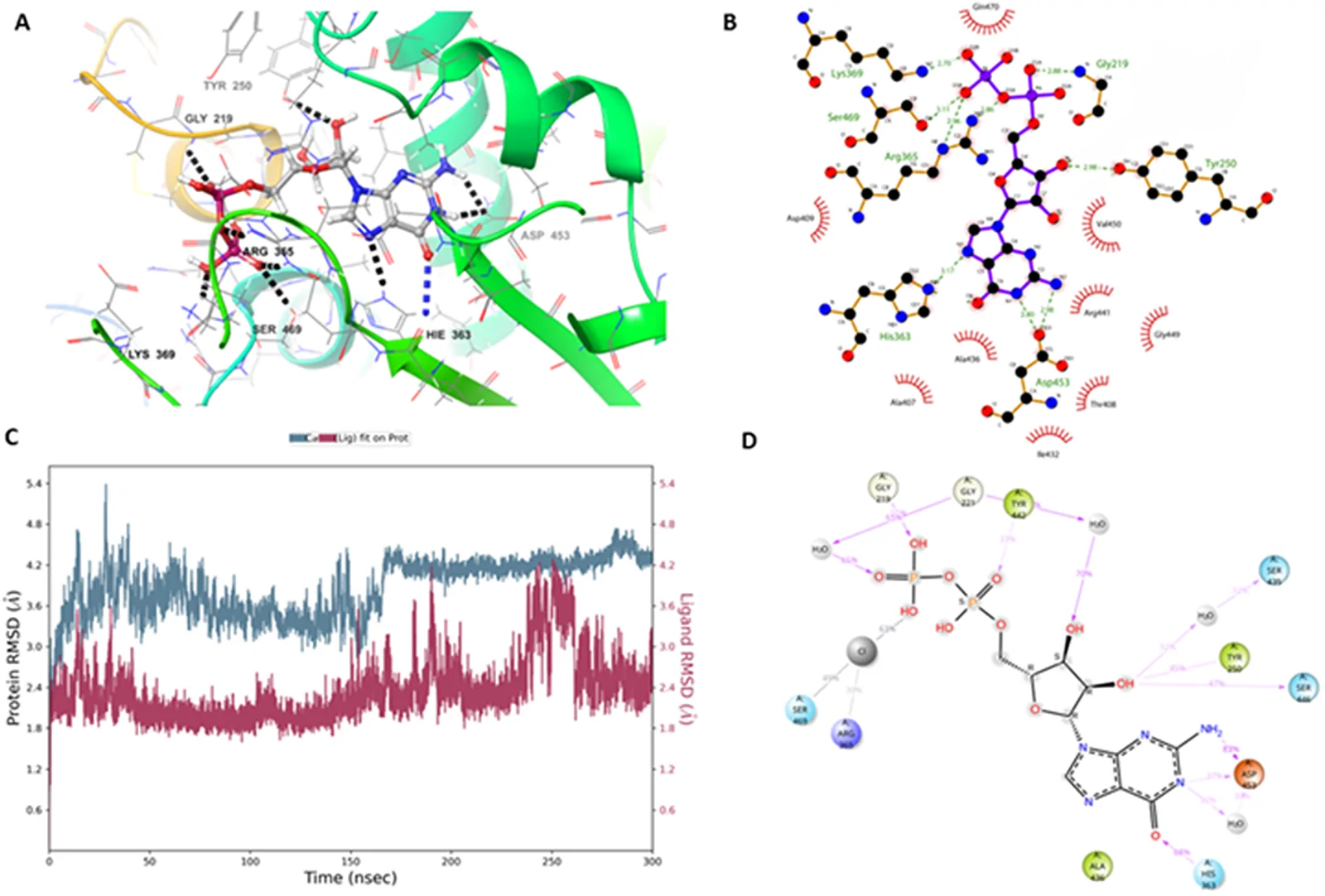
(A) The binding pose of GDP in the active site of human FUT8 obtained from molecular docking. Black dashed lines indicate hydrogen bonds, and blue dashed lines denote aromatic hydrogen bonds. (B) Two-dimensional LigPlot interaction map of the GDP–FUT8 complex showing hydrogen bonds and distances. (C) Root mean square deviation (RMSD) profiles of the FUT8 protein backbone (Cα, blue) and GDP fitted on FUT8 (red-brown) over the 300 ns molecular dynamics simulation. (D) Two-dimensional summary of GDP–FUT8 interactions from the MD trajectory, with percentages indicating the fraction of simulation frames in which each contact was maintained.

The stability of the GDP–enzyme complex was evaluated by molecular dynamics simulations. Protein backbone and ligand RMSD profiles were analyzed over a 300 ns trajectory (Fig 11C). During the initial phase of the simulation (0–40 ns), both RMSD values increased, reflecting relaxation from the docked conformation and equilibration of the solvent and side chains. In this interval, the protein RMSD increased to approximately 3.0–4.0 Å, while the ligand RMSD fluctuated within ~1.8–3.0 Å. After equilibration (40–170 ns), the protein RMSD reached a plateau and fluctuated around a stable mean of approximately 3.6 Å, indicating the absence of major conformational rearrangements. During this period, the ligand RMSD remained consistently lower than the protein RMSD and showed limited fluctuations, suggesting stable accommodation of GDP within the binding pocket. In the later stage of the simulation (170–300 ns), transient ligand RMSD fluctuations (~2.0–4.0 Å) were observed; however, these variations repeatedly returned to baseline values, indicating localized conformational adjustments of GDP rather than ligand dissociation. Throughout this phase, the protein RMSD remained stable at approximately 4.0 Å.

Protein–ligand interaction analysis showed polar anchoring of GDP within the FUT8 active site (Fig 11D). The guanine base maintained hydrogen-bond interactions with Asp453 (up to ~83% occupancy) and His363 (~68%), consistent with the docking results, while the ribose hydroxyl groups retained polar contacts with Tyr250 (~45%) and Ser446 (~47%). The phosphate groups displayed sustained contacts with Gly219 (up to ~65%), Tyr442, Arg365, and Ser469, together with frequent water-mediated bridges, forming an extended hydrogen-bonding network around the diphosphate moiety. Additional auxiliary contacts mediated through water molecules were observed with Gly221 and Ser435, contributing to ligand positioning within the binding pocket. Several docking-predicted interactions were preserved during simulation, although some transitioned to water-mediated contacts. Consistent with these persistent interactions, the total number of protein–ligand contacts remained high throughout the simulation, indicating a dense and sustained interaction network. Time-resolved contact maps and interaction histograms supporting these occupancies are provided in the Supplementary Information (Fig S4 and S5 in S1 File).

Post-MD MM/GBSA analysis of 201 frames extracted from the trajectory yielded an average binding free energy of −59.66 ± 5.90 kcal/mol, with values ranging from −79.64 to −44.23 kcal/mol, consistent with favorable binding during the simulation. The corresponding no-strain binding free energy was −66.23 ± 6.07 kcal/mol (range: −86.13 to −50.75 kcal/mol), showing the strong intrinsic affinity of GDP toward FUT8 after removal of ligand strain contributions. These results indicate a stable energetic profile throughout the trajectory. Hydrogen-bond analysis showed that GDP maintained approximately 2–6 hydrogen bonds with FUT8 throughout most of the 300 ns trajectory, with transient fluctuations but no prolonged loss of polar contacts, further supporting sustained ligand binding under dynamic conditions (Fig S6 in S1 File).

#### 2-Fluorofucose (2FF).

Docking analysis yielded a docking score of −5.378 and a binding free energy of −20.19 kcal/mol for 2FF bound to FUT8. The docking pose revealed a limited interaction profile, primarily involving hydrogen bonds with His363 and Asp453 (Fig 12A). A two-dimensional LigPlot interaction map showing hydrogen bonds together with bond distances is presented in Fig 12B.

**Fig 12:**
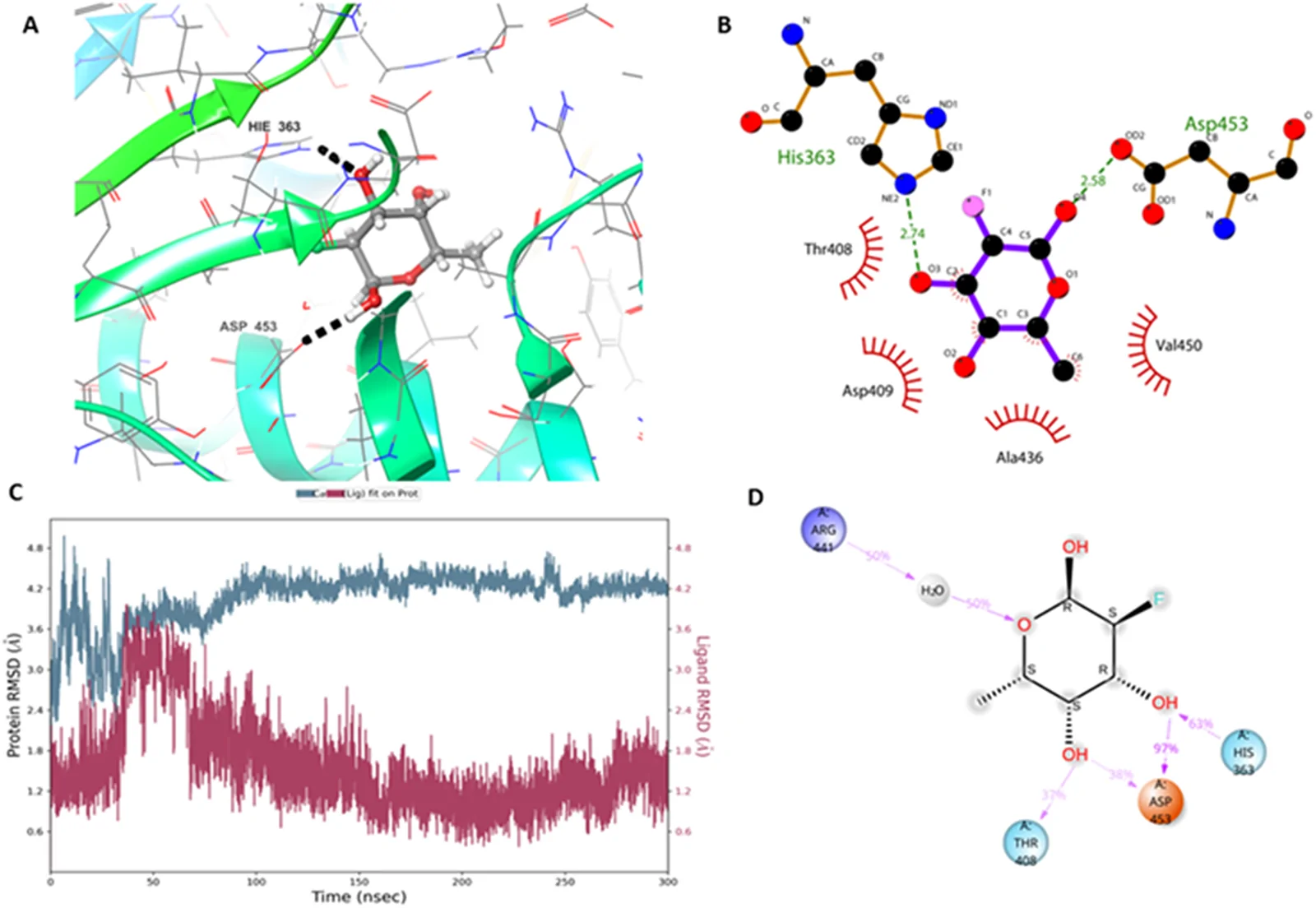
(A) Binding pose of 2-fluorofucose (2FF) in the active site of human FUT8 obtained from molecular docking, highlighting key hydrogen-bond interactions (black dashed lines). (B) Two-dimensional LigPlot interaction map of the 2FF–FUT8 complex showing hydrogen bonds together with bond distances. (C) Root mean square deviation (RMSD) profiles of the FUT8 protein backbone (Cα, blue) and 2FF fitted on FUT8 (red-brown) over the 300 ns molecular dynamics simulation. (D) Two-dimensional summary of protein–ligand interactions observed during the MD trajectory, showing occupancies of more than 30% of the simulation time.

The protein backbone and ligand RMSD were analyzed over the 300 ns MD simulation to assess the dynamic stability of the FUT8–2FF complex (Fig 12C). During the initial phase (0–70 ns), both protein and ligand RMSD values increased, reflecting relaxation from the docked conformation and equilibration of the system. In this interval, the protein RMSD increased to approximately 3.0–4.0 Å, while the ligand RMSD increased to ~3.0–3.5 Å. Following equilibration, the protein RMSD stabilized around a mean value of approximately 4.2 Å, indicating the absence of major conformational rearrangements. On the other hand, the ligand RMSD gradually decreased and stabilized within ~1.0–2.0 Å, suggesting improved accommodation of 2FF within the binding pocket, with moderate fluctuations and no evidence of progressive drift or dissociation.

Protein–ligand contacts persisting for more than 30% of the simulation were summarized in a two-dimensional interaction map (Fig 12D). A dominant hydrogen bond with Asp453 was maintained for ~97% of the trajectory, while His363 showed moderate occupancy (~63%), consistent with docking. A hydrogen bond with Thr408 persisted for ~37% of the simulation, and Arg441 contributed through a water-mediated bridge (~50%). Time-resolved interaction histograms and contact timelines (Figs S7 and S8 in S1 File) indicated that, beyond His363 and Asp453, all remaining contacts were transient and displayed low occupancy, with total protein–ligand contacts fluctuating mainly between two and six throughout the 300 ns trajectory. These results indicate the absence of a dense or cooperative interaction network, with 2FF binding primarily stabilized by two dominant hydrogen bonds supplemented by intermittent polar and solvent-mediated contacts.

Post-MD MM/GBSA analysis of 201 trajectory frames yielded an average binding free energy of −30.98 ± 3.86 kcal/mol, with values ranging from −40.08 to −21.08 kcal/mol, indicating moderate but variable binding during the simulation. The corresponding no-strain binding free energy was −32.48 ± 3.87 kcal/mol (range: −41.77 to −22.89 kcal/mol). Hydrogen-bond monitoring revealed fluctuating interaction numbers, with intermittent hydrogen bonds maintained throughout the trajectory (Fig S9 in S1 File). Collectively, these results indicate engagement of 2FF within the FUT8 active site, dominated by Asp453- and His363-mediated hydrogen bonds and supplemented by transient polar and water-mediated interactions, consistent with its limited docking interactions and modest binding energetics.

Compound **1** exhibited a docking score of −5.421 and a binding free energy of −34.81 kcal/mol. In the docking pose, the ligand formed a hydrogen bond with Arg365, together with a π–cation interaction between the pyridone ring of Compound **1** and Arg365 (Fig 13A). A two-dimensional LigPlot interaction map showing hydrogen bonds together with bond distances is presented in Fig 13B.

**Fig 13:**
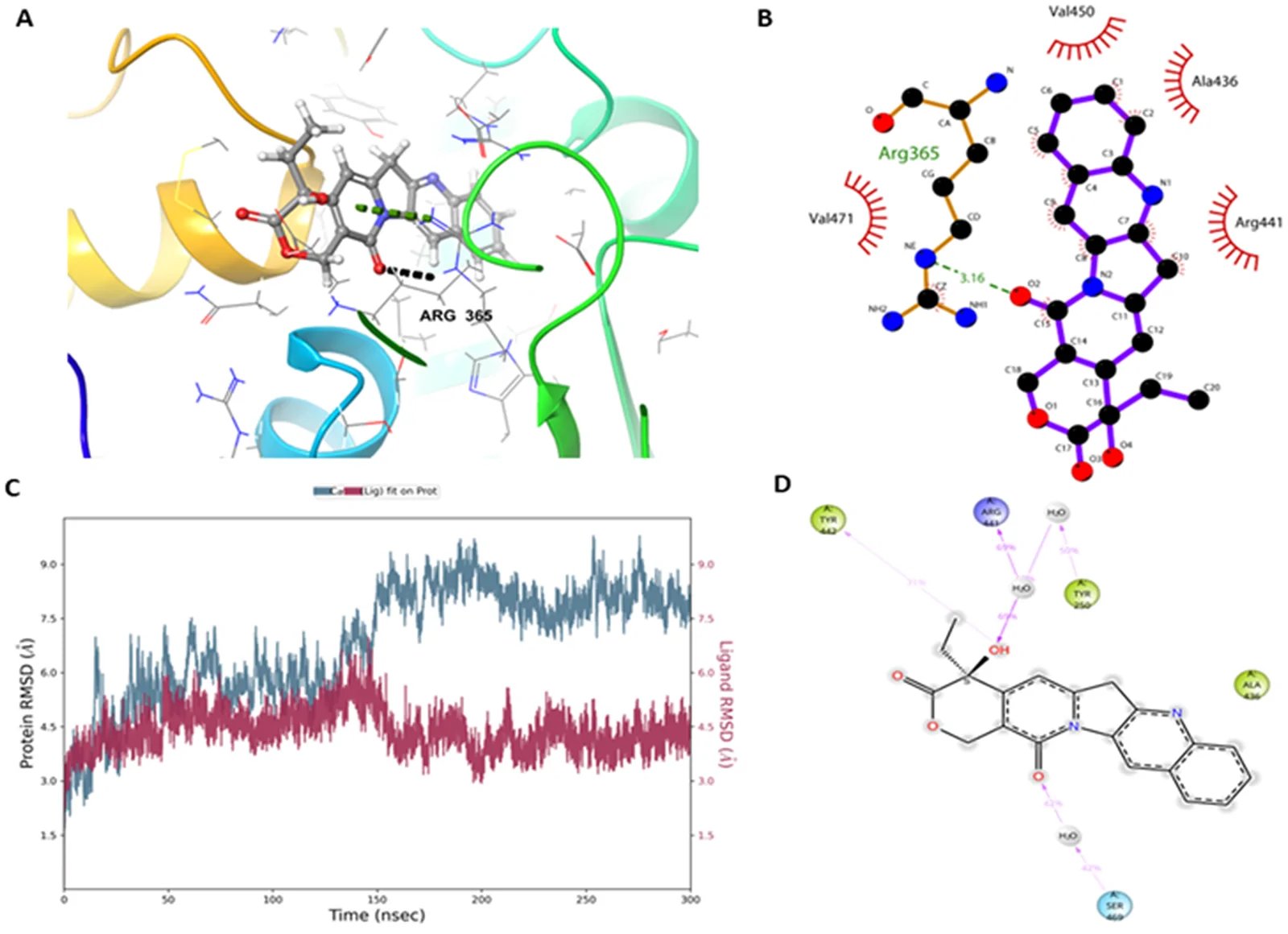
(A) Binding pose of compound 1 in the active site of human FUT8 obtained from molecular docking (black dashed lines indicate hydrogen bonds; green dashed line indicates π–cation interaction). (B) Two-dimensional LigPlot interaction map of compound **1**–FUT8 showing hydrogen-bond interactions and bond distances. (C) RMSD profiles of the FUT8 protein backbone (Cα, blue) and compound **1** fitted on FUT8 (red-brown) over the 300 ns molecular dynamics simulation. (D) Two-dimensional summary of protein–ligand interactions observed during the MD trajectory, showing occupancies of more than 30% of the simulation time.

The dynamic stability of the FUT8–Compound **1** complex was evaluated by monitoring protein backbone and ligand RMSD over a 300 ns MD trajectory (Fig 13C). During the initial phase (0–30 ns), both RMSD traces increased rapidly, reflecting relaxation from the docked conformation and equilibration of the system. In this interval, the protein RMSD increased from approximately 2.5 Å to 4.0–5.0 Å, while the ligand RMSD increased to ~4.0–4.5 Å. During the intermediate phase (30–150 ns), the protein RMSD continued to increase gradually and fluctuated around 5.0–6.5 Å, indicating ongoing conformational adjustment of FUT8. Over the same period, the ligand RMSD remained elevated and variable, reaching a local maximum near 140–150 ns (~5.5–6.0 Å), consistent with positional mobility of Compound **1** within the binding pocket. In the later stage of the simulation (150–300 ns), the protein RMSD reached a higher plateau (7.5–8.5 Å) and remained stable thereafter, suggesting adoption of an alternative protein conformation. Following this transition, the ligand RMSD decreased and stabilized at ~3.5–4.5 Å, with moderate fluctuations but no evidence of progressive drift or dissociation.

Protein–ligand interactions persisting for more than 30% of the simulation were summarized in a two-dimensional interaction map (Fig 13D). In contrast to the docking pose, only one direct hydrogen bond with Tyr442 was maintained during MD, with an occupancy of ~31%. Most remaining interactions were solvent-mediated, predominantly involving Arg441 and Tyr250 through bridging water molecules (up to ~69% occupancy), while Ser469 participated intermittently *via* water-mediated contacts (~42%). Hydrophobic contributions, including contacts with Ala436, were observed in the interaction frequency histogram (Fig S10 in S1 File). Time-resolved contact timelines (Fig S11 in S1 File) showed that total protein–ligand contacts fluctuated primarily between 1 and 6 throughout the trajectory.

Post-MD MM/GBSA analysis of 201 trajectory frames yielded an average binding free energy of −46.45 ± 3.61 kcal/mol, with values ranging from −56.65 to −35.97 kcal/mol, indicating significantly stronger binding than 2FF. The corresponding no-strain binding free energy was −47.56 ± 3.64 kcal/mol (range: −59.47 to −37.45 kcal/mol). Hydrogen-bond analysis revealed fluctuating interaction numbers throughout the trajectory, consistent with the dynamic contact profile observed in the interaction timelines (Fig S12 in S1 File). Collectively, these results indicate stable but flexible engagement of compound **1** within the FUT8 active site, driven by favorable binding energetics and maintained through a combination of transient direct hydrogen bonds, water-mediated interactions, and hydrophobic contacts.

Compound **2** showed a docking score of −4.697 and a binding free energy of −43.83 kcal/mol. In the docking pose, the ligand formed two H-bonds with Arg441 and Gln470 residues of FUT8, while π-cation interaction was established between the phenyl ring of the styrene moiety of compound **2** and Arg365. An aromatic hydrogen bond was also observed between the styrene ring of the ligand and the Glu373 residue of the enzyme (Fig 14A).). A two-dimensional LigPlot interaction map showing hydrogen bonds together with bond distances is presented in Fig 14B.

**Fig 14:**
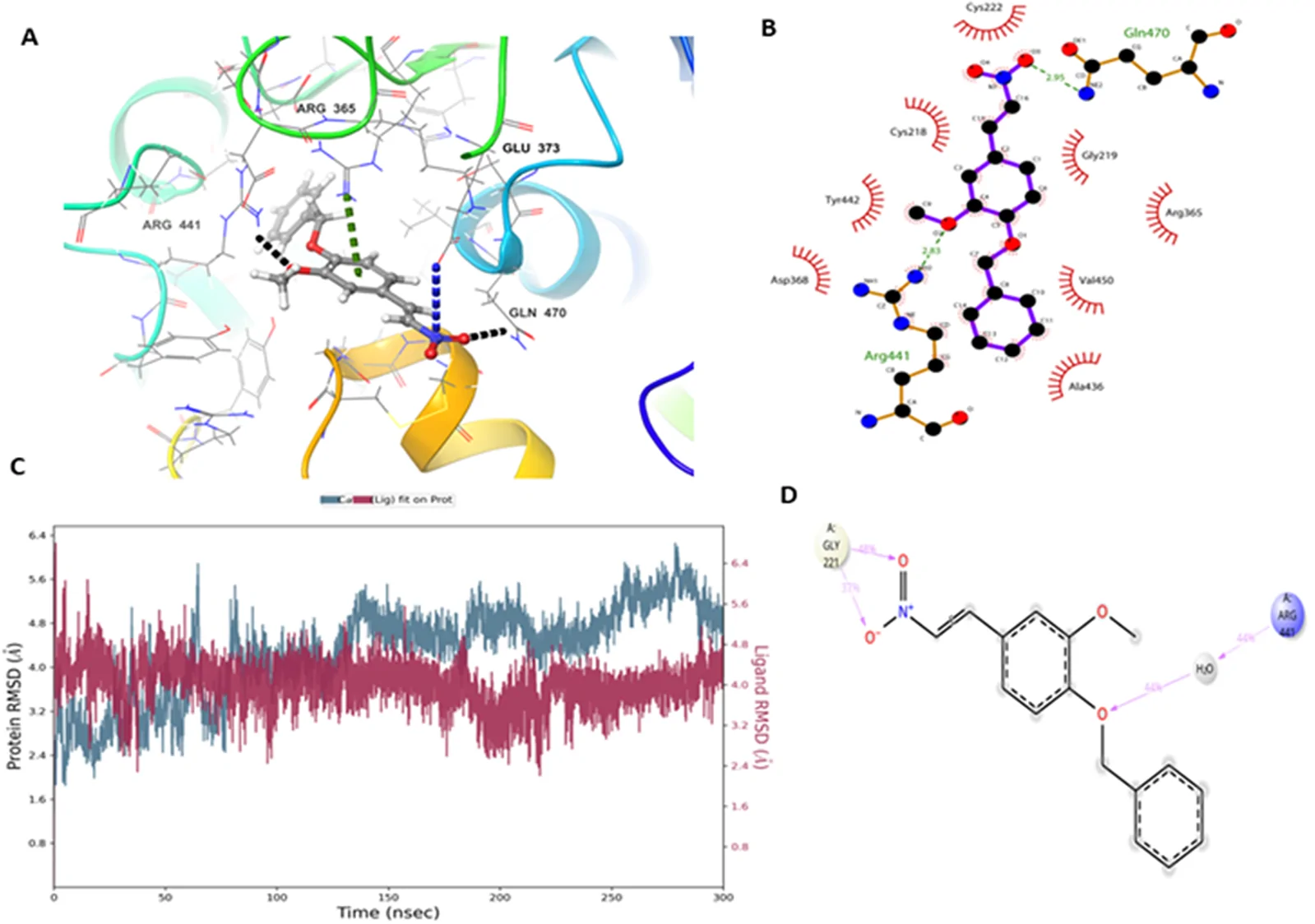
(A) Binding pose of compound 2 in the active site of human FUT8 obtained from molecular docking (black dashed lines indicate hydrogen bonds; blue dashed line indicates aromatic hydrogen bond; green dashed line indicates π–cation interaction). (B) Two-dimensional LigPlot interaction map of compound **2**–FUT8 showing hydrogen-bond interactions and bond distances. (C) RMSD profiles of the FUT8 protein backbone (Cα, blue) and compound **2** fitted on FUT8 (red-brown) over the 300 ns molecular dynamics simulation. (D) Two-dimensional summary of protein–ligand interactions observed during the MD trajectory, showing occupancies of more than 30% of the simulation time.

The dynamic stability of the Compound 2–FUT8 complex was evaluated by analyzing the protein backbone and ligand RMSD over a 300 ns MD simulation (Fig 14C). The protein RMSD increased gradually from approximately 2.0–2.5 Å at the beginning to ~5.5–6.0 Å toward the end of the trajectory, indicating progressive conformational adjustment without abrupt structural transitions. On the other hand, the ligand RMSD remained relatively stable throughout the simulation, fluctuating mainly within the range of ~3.5–4.2 Å. A transient decrease in ligand RMSD was observed around 190–220 ns; however, the ligand then returned to its previous fluctuation range, with no evidence of drift or dissociation, indicating continued association of compound **2** with the FUT8 binding site.

Protein–ligand interaction analysis during the MD simulation revealed that compound **2** binding was dominated by transient and water-mediated contacts rather than stable direct hydrogen bonding (Fig 14D). While the docking pose indicated stabilizing interactions, including a π–cation interaction between the phenyl ring of the styrene moiety of compound **2** and Arg365 and hydrogen bonds involving Arg441 and Gln470, these contacts were not persistently maintained under dynamic conditions. During the simulation, the most persistent interactions involved Gly221, which formed hydrogen bonds with the nitro group of the ligand for approximately 48% of the simulation time, along with an additional contact maintained for ~37%. Water-bridged interactions involving Arg441 were also prominent, with occupancies of ~44%, highlighting the contribution of solvent-mediated stabilization. Intermittent hydrophobic contacts involving Val471 were observed in the interaction frequency histogram (Fig S13 in S1 File), indicating that nonpolar interactions contribute transiently to ligand stabilization. Other contacts appeared intermittently and were not retained for the majority of the trajectory. Quantitative protein–ligand contact frequencies and time-resolved interaction profiles are provided in the Supplementary Information (Figs S13 and S14 in S1 File).

Post-MD MM/GBSA analysis of 201 trajectory frames yielded an average binding free energy of −41.69 ± 3.72 kcal/mol, with values ranging from −53.68 to −32.65 kcal/mol, indicating favorable binding of compound **2** to FUT8. The corresponding no-strain binding free energy was −43.00 ± 3.93 kcal/mol (range: −56.21 to −35.68 kcal/mol). Hydrogen-bond analysis revealed fluctuating interaction numbers throughout the trajectory, with predominantly one to two hydrogen bonds maintained at any given time and occasional transient increases (Fig S15 in S1 File), consistent with the dynamic contact profile observed in the interaction timelines. Overall, these results indicate persistent but flexible engagement of compound **2** within the FUT8 active site, maintained by a combination of transient hydrogen bonds, water-mediated interactions, and hydrophobic contacts.

The compound **3** showed a docking score of −5.940 with a binding free energy of −42.52 kcal/mol. In the docking pose, the ligand formed a hydrogen bond between the OH group of the indolinone ring and Tyr250. Moreover, an aromatic hydrogen bond was observed between the aromatic ring of the indolinone scaffold and Asp453 (Fig 15A). A two-dimensional LigPlot interaction map showing a hydrogen bond, along with bond distance, is presented in Fig 15B.

**Fig 15:**
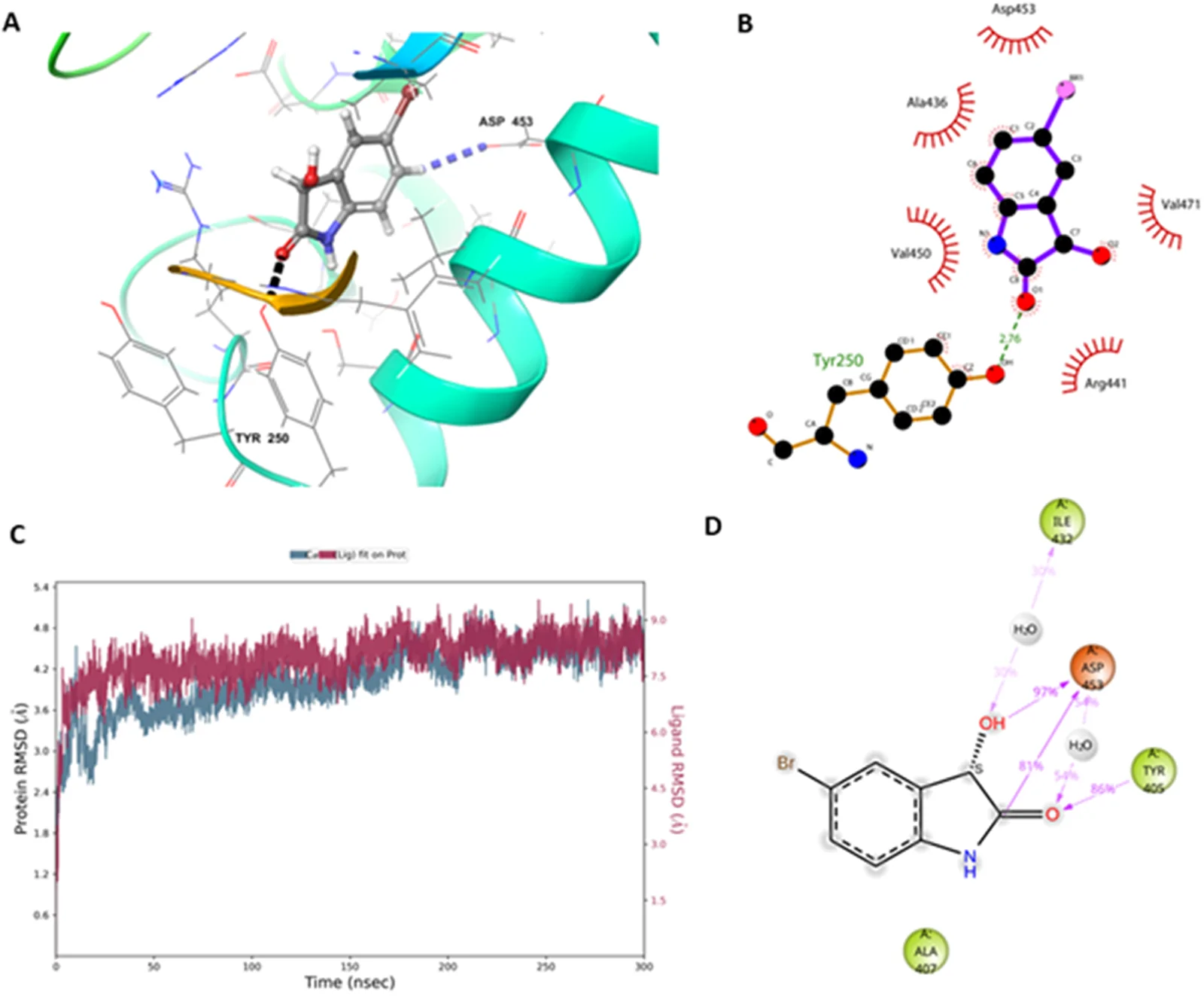
(A) Binding pose of compound 3 in the active site of human FUT8 obtained from molecular docking (black dashed lines indicate hydrogen bonds; blue dashed line indicates aromatic hydrogen bond). (B) Two-dimensional LigPlot interaction map of compound **3**–FUT8 showing hydrogen-bond interactions and bond distances. (C) RMSD profiles of the FUT8 protein backbone (Cα, blue) and compound **3** fitted on FUT8 (red-brown) over the 300 ns molecular dynamics simulation. (D) Two-dimensional summary of protein–ligand interactions observed during the MD trajectory, showing occupancies of more than 30% of the simulation time.

The dynamic stability of the compound **3**–FUT8 complex was evaluated by monitoring the protein backbone and ligand RMSD over a 300 ns MD simulation (Fig 15C). Following a brief initial equilibration phase, the protein RMSD increased from approximately 2.5 Å to ~3.5–4.0 Å during the early stage of the simulation, then gradually increased, stabilizing around ~4.2–4.6 Å for the remainder of the trajectory. This behavior indicates moderate conformational adjustment of the protein without large-scale structural rearrangements. On the other hand, the ligand RMSD increased rapidly during the early phase of the simulation and then fluctuated within a broader range of ~8.0–9.0 Å throughout the trajectory. Despite these fluctuations, no sustained drift or progressive increase in ligand RMSD was observed, indicating that compound **3** remained bound within the FUT8 active site while undergoing conformational rearrangements.

Protein–ligand interaction analysis during the MD simulation indicated that compound **3** binding was primarily stabilized by persistent hydrogen bonding and water-mediated interactions rather than rigid direct contacts (Fig 15D). The most dominant interactions involved Asp453, which formed stable hydrogen bonds with both the hydroxyl and carbonyl groups of the ligand, showing high occupancies of approximately 97% and 81%, respectively. Notably, Asp453, identified as a key interaction partner in the docking pose, remained the principal anchoring residue throughout the MD simulation, indicating good agreement between docking predictions and dynamic behavior. Additional solvent-bridged interactions involving Asp453 further stabilized the ligand throughout the trajectory. Contacts with Tyr405 were also prominent, with occupancies of ~86%, supporting indirect but persistent ligand engagement within the binding pocket. In contrast, the interaction involving Tyr250 observed in the docking pose was not maintained under dynamic, solvent-exposed conditions. Weaker and transient interactions were observed with Ile432 (~30%) and surrounding nonpolar residues, including Ala407, which contributed modestly to ligand positioning. Quantitative protein–ligand contact frequencies and time-resolved interaction profiles are provided in the Supplementary Information (Figs S16 and S17 in S1 File).

Post-MD MM/GBSA analysis of 201 trajectory frames yielded an average binding free energy of −36.41 ± 3.89 kcal/mol, with values ranging from −47.88 to −25.06 kcal/mol. The corresponding no-strain binding free energy was −37.18 ± 3.85 kcal/mol (range: −48.15 to −25.63 kcal/mol). Hydrogen-bond analysis revealed fluctuating interaction numbers throughout the trajectory, with mainly two to three hydrogen bonds maintained at any given time and occasional transient increases (Fig S18 in S1 File). Together, these results indicate stable but flexible engagement of compound **3** within the FUT8 active site, dominated by strong polar interactions through Asp453 and supported by solvent-mediated and weak hydrophobic interactions, consistent with the docking-predicted binding mode.

Compound **4** showed a docking score of −5.326 with a binding free energy of −21.60 kcal/mol. In the docking pose, the ligand formed hydrogen-bond interactions with Tyr250 and Asp453 residues of FUT8, positioning the molecule within the catalytic pocket (Fig 16A). A two-dimensional LigPlot interaction map showing hydrogen bonds together with bond distances is presented in Fig 16B.

**Fig 16:**
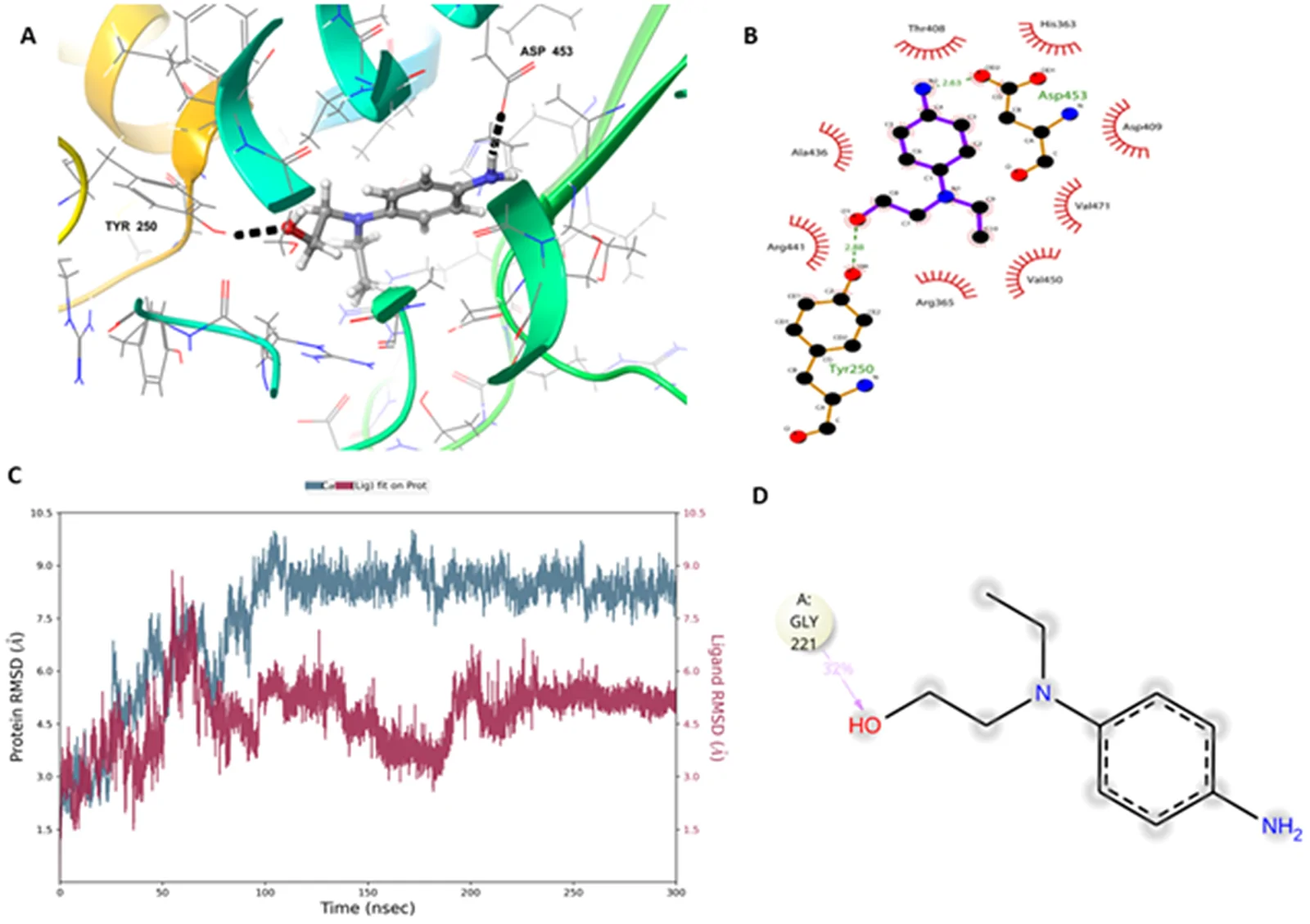
(A) Binding pose of compound 4 in the active site of human FUT8 obtained from molecular docking (black dashed lines indicate hydrogen). (B) Two-dimensional LigPlot interaction map of compound **4**–FUT8 showing hydrogen-bond interactions and bond distances. (C) RMSD profiles of the FUT8 protein backbone (Cα, blue) and compound **4** fitted on FUT8 (red-brown) over the 300 ns molecular dynamics simulation. (D) Two-dimensional summary of protein–ligand interactions observed during the MD trajectory, showing occupancies of more than 30% of the simulation time.

The dynamic stability of the compound **4**–FUT8 complex was evaluated by analyzing the protein backbone and ligand RMSD over a 300 ns MD simulation (Fig 16C). During the initial phase of the simulation (0–70 ns), both the protein and ligand RMSD values increased significantly, reflecting relaxation from the docked conformation and equilibration of the system. In this interval, the protein RMSD increased from approximately 2.5 Å to ~6.5–7.5 Å, while the ligand RMSD increased to ~5.5–7.5 Å. Following equilibration, the protein RMSD continued to increase and stabilized at ~8.0–9.0 Å for the remainder of the trajectory, indicating significant conformational rearrangement of the protein. On the other hand, ligand RMSD decreased after ~80–100 ns and fluctuated within a narrower range of ~4.5–5.5 Å during the later stages of the simulation. Despite the molecule’s small size contributing to some RMSD variability in the complex, no sustained drift or progressive increase in ligand RMSD was observed, suggesting that the ligand remained within the binding pocket while adopting a dynamically adjusted binding pose.

Protein–ligand interaction analysis during the MD simulation revealed a predominantly dynamic interaction profile characterized by transient hydrogen bonds and water-mediated contacts (Fig 16D). The most persistent direct interaction involved Gly221, which formed hydrogen bonds with the ligand for approximately 32% of the simulation time. Interactions with Tyr250 and Asp453, identified in the docking pose, were observed intermittently but were not consistently maintained throughout the trajectory. Several water-bridged interactions contributed to ligand stabilization, indicating solvent-assisted binding under dynamic conditions. Weak and transient contacts with surrounding residues, including Gln470 and Val471, were also detected in the interaction frequency histogram (Fig S19 in S1 File), suggesting minor contributions from nonpolar interactions. Quantitative protein–ligand contact frequencies and time-resolved interaction profiles are provided in the Supplementary Information (Figs S19 and S20 in S1 File).

Post-MD MM/GBSA analysis of 201 trajectory frames yielded an average binding free energy of −32.96 ± 4.95 kcal/mol, with values ranging from −46.93 to −20.27 kcal/mol. The corresponding no-strain binding free energy was −34.55 ± 4.74 kcal/mol (range: −49.46 to −21.52 kcal/mol). Hydrogen-bond analysis revealed fluctuating interaction numbers throughout the trajectory, with predominantly one to three hydrogen bonds maintained at any given time and occasional transient increases (Fig S21 in S1 File). Together, these results indicate stable but flexible engagement of compound **4** within the FUT8 active site, maintained primarily through dynamic hydrogen bonding and solvent-mediated interactions, with minor contributions from weak hydrophobic contacts, consistent with the observed ligand mobility during the simulation.

Compound **5** showed a docking score of −6.173 with a binding free energy of −11.63 kcal/mol, indicating comparatively weak binding to FUT8. In the docking pose, the ligand formed a hydrogen-bond interaction between its amino (NH_2_) group and the Asp453 residue of the enzyme, positioning the molecule near the catalytic region (Fig 17A). No additional stabilizing polar or hydrophobic interactions were observed in the docked complex. A two-dimensional LigPlot interaction map showing hydrogen bonds together with bond distances is presented in Fig 17B.

**Fig 17:**
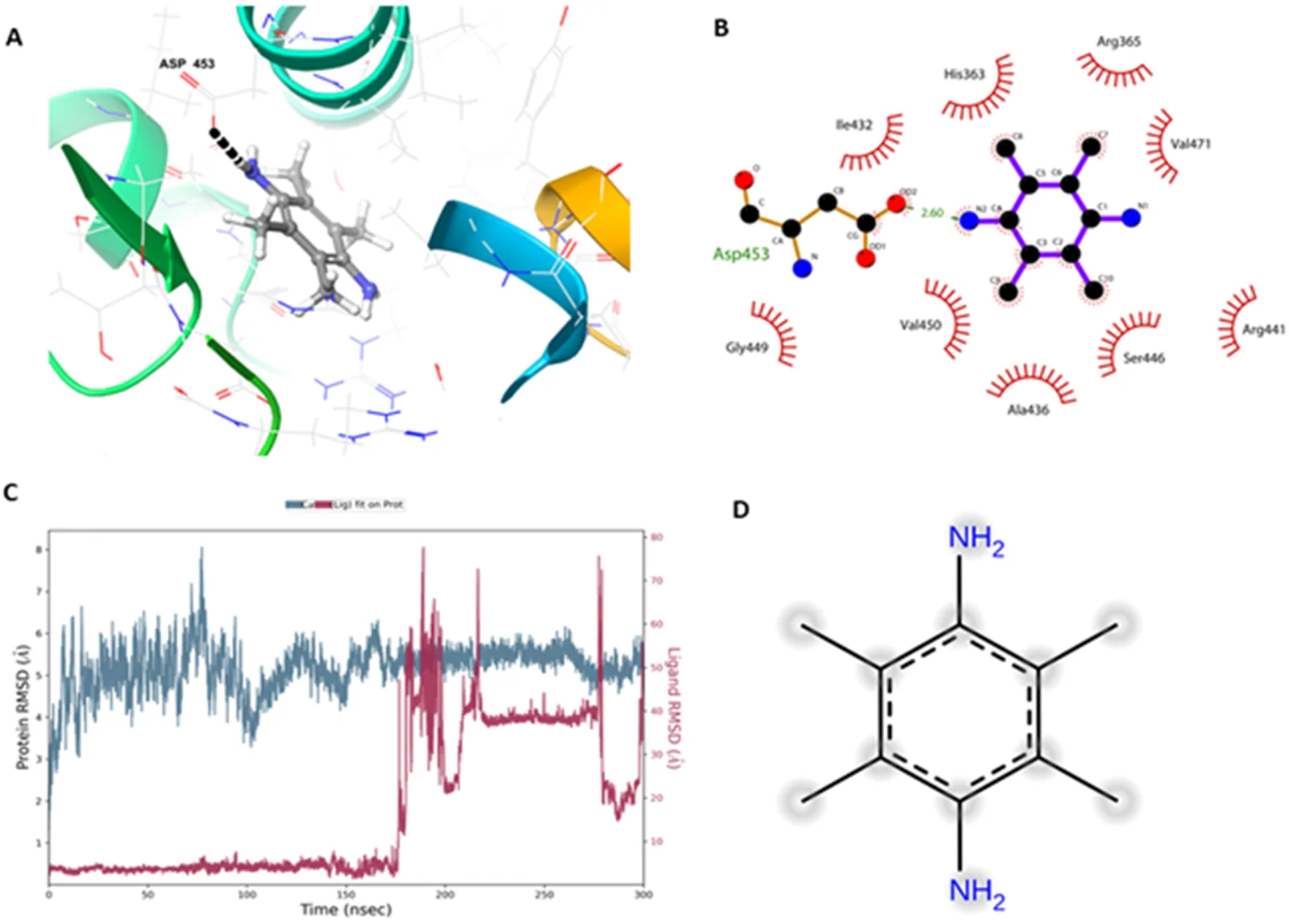
(A) Binding pose of compound 5 in the active site of human FUT8 obtained from molecular docking (black dashed lines indicate hydrogen bonds). (B) Two-dimensional LigPlot interaction map of compound **5**–FUT8 showing hydrogen-bond interactions and bond distances. (C) RMSD profiles of the FUT8 protein backbone (Cα, blue) and compound **5** fitted on FUT8 (red-brown) over the 300 ns molecular dynamics simulation. (D) Two-dimensional summary of protein–ligand interactions observed during the MD trajectory, showing occupancies of more than 30% of the simulation time.

The dynamic behavior of the compound **5**–FUT8 complex evaluated over a 300 ns MD simulation (Fig 17C), showed that during the initial phase of the simulation (0–40 ns), the protein RMSD increased from approximately 2.0 Å to ~4.5–5.0 Å, reflecting relaxation from the docked conformation, and then fluctuated within the range of ~4.5–6.0 Å for the remainder of the trajectory, indicating overall structural stability of the protein. On the other hand, the ligand RMSD remained very low during the early and intermediate stages of the simulation, fluctuating below ~1.0 Å up to approximately 160–170 ns, suggesting transient retention of the initial docking pose. However, a sharp increase in ligand RMSD was observed after this point, followed by sustained high fluctuations, indicating displacement of compound **5** from its initial binding position. Despite intermittent reductions in RMSD, no long-term re-stabilization within the binding pocket was observed. These results suggest that compound **5** does not maintain a stable binding mode under dynamic conditions, consistent with its weak docking score and limited interaction profile.

Protein–ligand interaction analysis revealed a predominantly transient interaction profile during the MD simulation (Fig 17D). Hydrogen-bond interactions involving Asp453, identified in the docking pose, were not persistently maintained throughout the trajectory. Instead, short-lived contacts and intermittent water-mediated interactions dominated the interaction landscape. The interaction timeline and histogram analyses (Figs S22 and S23 in S1 File) showed low overall contact occupancies, with no single residue forming stable interactions for a significant fraction of the simulation time.

Post-MD MM/GBSA analysis of 201 trajectory frames yielded an average binding free energy of −24.93 ± 8.20 kcal/mol, with values ranging from −43.75 to 0.26 kcal/mol, reflecting significant energetic fluctuations during the simulation. The corresponding no-strain binding free energy was −25.72 ± 8.43 kcal/mol (range: −45.77 to 0.15 kcal/mol). Hydrogen-bond analysis revealed highly fluctuating interaction numbers throughout the trajectory, with mainly zero to two hydrogen bonds maintained at any given time and frequent loss of polar contacts (Fig S24 in S1 File). Collectively, these results indicate weak and unstable engagement of compound **5** within the FUT8 active site, characterized by pronounced energetic variability, transient hydrogen bonding, and limited solvent-assisted stabilization, consistent with the observed ligand displacement during the simulation.

Compound **6** showed a docking score of −5.310 with a binding free energy of −23.90 kcal/mol. In the docking pose, Asp453 formed a weak C–H···O interaction (aromatic hydrogen bond) with the halogen-substituted aromatic carbon of the lignad, while Ser446 engaged the phenolic hydroxyl group through a conventional hydrogen bond, positioning the molecule within the catalytic pocket (Fig 18A). No additional stabilizing polar or hydrophobic interactions were observed in the docked complex. A two-dimensional LigPlot interaction map showing hydrogen bonds together with bond distances is presented in Fig 18B.

**Fig 18:**
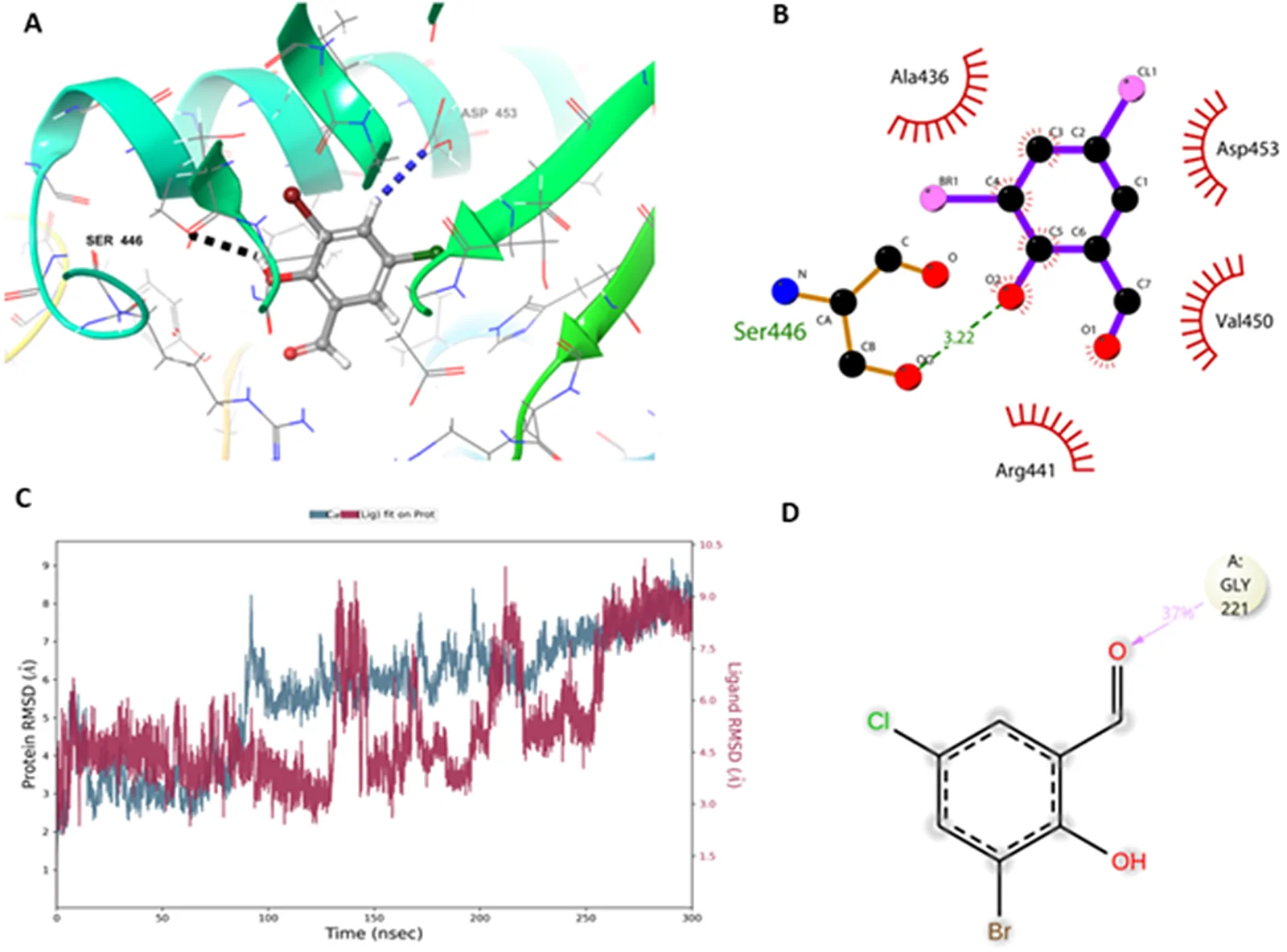
(A) Binding pose of compound 6 in the active site of human FUT8 obtained from molecular docking (black dashed lines indicate hydrogen bonds; blue dashed line indicates aromatic hydrogen bond). (B) Two-dimensional LigPlot interaction map of compound **6**–FUT8 showing hydrogen-bond interactions and bond distances. (C) RMSD profiles of the FUT8 protein backbone (Cα, blue) and compound **6** fitted on FUT8 (red-brown) over the 300 ns molecular dynamics simulation. (D) Two-dimensional summary of protein–ligand interactions observed during the MD trajectory, showing occupancies of more than 30% of the simulation time.

The dynamic stability of the compound **6**–FUT8 complex was evaluated by monitoring the protein backbone and ligand RMSD over a 300 ns MD simulation (Fig 18C). During the initial phase of the simulation (0–80 ns), both RMSD traces increased, consistent with relaxation from the docked conformation and equilibration of the system. In this interval, the protein RMSD increased from approximately 2.0 Å to ~3.5–4.0 Å, while the ligand RMSD fluctuated around ~3**.**0–4.5 Å. During the intermediate and later stages of the simulation, the protein RMSD gradually increased and fluctuated mainly within the range of ~5.5–7.0 Å, rising to ~8.0 Å after 240 ns, indicating progressive conformational adjustment of the protein. On the other hand, the ligand RMSD showed broad fluctuations throughout the trajectory, ranging from ~3.0 Å to ~8.0–9.0 Å after ~130 ns. Despite these fluctuations, no sustained drift or complete dissociation was observed, indicating that compound **6** remained associated with the FUT8 binding site, while undergoing significant positional rearrangements.

Protein–ligand interaction analysis during the MD simulation revealed a predominantly transient interaction profile (Fig 18D). The aromatic hydrogen bond with Asp453 identified in the docking pose was not persistently maintained under dynamic conditions. Instead, the most frequent interaction involved Gly221, which formed hydrogen bonds for approximately 37% of the simulation time. Additional contacts with Tyr220, Tyr250, Ser446, and Val471 were observed intermittently, along with water-mediated interactions, indicating solvent-assisted stabilization (Figs S25 in S1 File). No single residue formed stable interactions for most of the trajectory, and the total number of contacts fluctuated mainly between 2 and 5 throughout the simulation (Fig S26 in S1 File). Quantitative protein–ligand contact frequencies and time-resolved interaction profiles are provided in the Supplementary Information (Figs S25 and S26 in S1 File).

Post-MD MM/GBSA analysis of 201 trajectory frames yielded an average binding free energy of −32.72 ± 6.23 kcal/mol, with values ranging from −48.06 to −18.62 kcal/mol, indicating significant energetic fluctuations during the simulation. The corresponding no-strain binding free energy was −33.10 ± 6.28 kcal/mol (range: −49.38 to −20.57 kcal/mol). Hydrogen-bond analysis revealed predominantly zero to one hydrogen bonds throughout the trajectory, with only transient increases to two interactions and frequent loss of polar contacts (Fig S27 in S1 File). Collectively, these results indicate weak, highly dynamic engagement of compound **6** within the FUT8 active site, characterized by significant energetic fluctuations and maintained by transient hydrogen bonds and solvent-mediated interactions, in agreement with its limited docking interactions and pronounced ligand mobility during molecular dynamics simulation.

Compound **7** showed a docking score of −7.286 with a binding free energy of −54.31 kcal/mol. In the docking pose, the hydroxyl group of the ligand formed a hydrogen bond with Asp453 of FUT8, positioning the molecule within the catalytic pocket (Fig 19A). A two-dimensional LigPlot interaction map showing hydrogen bonds together with bond distances is presented in Fig 19B.

**Fig 19:**
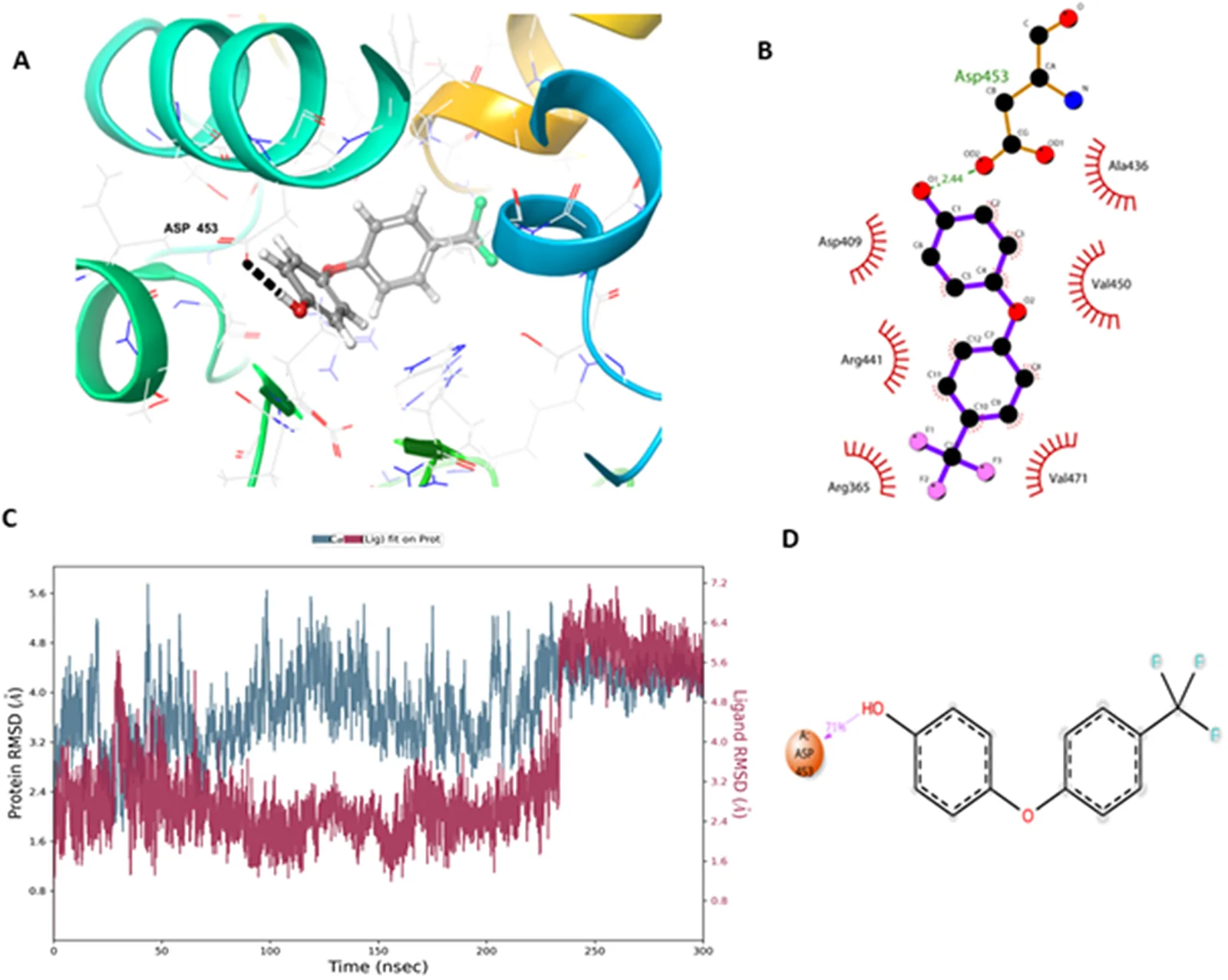
(A) Binding pose of compound 7 in the active site of human FUT8 obtained from molecular docking (black dashed lines indicate hydrogen bonds). (B) Two-dimensional LigPlot interaction map of compound **7**–FUT8 showing hydrogen-bond interactions and bond distances. (C) RMSD profiles of the FUT8 protein backbone (Cα, blue) and compound **7** fitted on FUT8 (red-brown) over the 300 ns molecular dynamics simulation. (D) Two-dimensional summary of protein–ligand interactions observed during the MD trajectory, showing occupancies of more than 30% of the simulation time.

The dynamic stability of the compound **7**–FUT8 complex was evaluated over a 300 ns MD simulation (Fig 19C). During the initial equilibration phase (0–60 ns), both RMSDs increased, consistent with relaxation from the docked conformation. In this interval, the protein RMSD increased from approximately 2.0 Å to ~3.5–4.0 Å, while the ligand RMSD fluctuated around ~2.0–3.5 Å. During the intermediate stage (90–230 ns), the protein RMSD remained mainly within ~3.0–4.5 Å, indicating moderate conformational adjustment without major structural rearrangements. Over the same period, the ligand RMSD remained relatively low and stable, suggesting sustained accommodation of compound **7** within the binding pocket. After approximately 230 ns, a shift in ligand RMSD was observed, followed by stabilization in the range of ~4.5–5.5 Å during the remainder of the simulation. Despite this late-stage positional adjustment, no progressive drift or dissociation was detected, indicating sustained association of compound **7** with the FUT8 active site.

Protein–ligand interaction analysis showed that compound **7** binding was primarily stabilized by a persistent hydrogen-bond interaction with Asp453, with an occupancy of approximately 71% over the trajectory (Fig 19D). This residue, identified in the docking pose as the principal interaction partner, remained the dominant anchoring site throughout the simulation. Additional interactions were observed with Ser431, Ala436, Gly449, and Val450; however, these contacts were weaker and more transient, with occupancies below 20%, contributing modestly to ligand positioning within the binding pocket. The total number of protein–ligand contacts fluctuated throughout the simulation, with increased interaction density observed after approximately 200 ns (Fig S28 in S1 File), coinciding with the late-stage ligand repositioning observed in the RMSD analysis. Quantitative contact frequencies and time-resolved interaction profiles are provided in the supplementary information (Figs S28 and S29 in S1 File).

Post-MD MM/GBSA analysis of 201 trajectory frames yielded an average binding free energy of −41.60 ± 4.41 kcal/mol, with values ranging from −54.43 to −30.00 kcal/mol. The corresponding no-strain binding free energy was −43.27 ± 4.57 kcal/mol (range: −55.58 to −30.11 kcal/mol). Hydrogen-bond analysis revealed fluctuating interaction numbers throughout the trajectory, typically ranging between one and two hydrogen bonds, with occasional transient increases (Fig S30 in S1 File). Together, these results indicate stable but flexible engagement of compound 7 within the FUT8 active site, dominated by persistent polar anchoring through Asp453 and supported by transient auxiliary contacts, consistent with the docking-predicted binding mode.

Compound **8** showed a docking score of −4.990 with a binding free energy of −30.68 kcal/mol. In the docking pose, the hydroxyl groups of the ligand formed hydrogen-bond interactions with Gly221 and Tyr250 of FUT8 (Fig 20A). A two-dimensional LigPlot interaction map showing hydrogen bonds together with bond distances is presented in Fig 20B.

**Fig 20:**
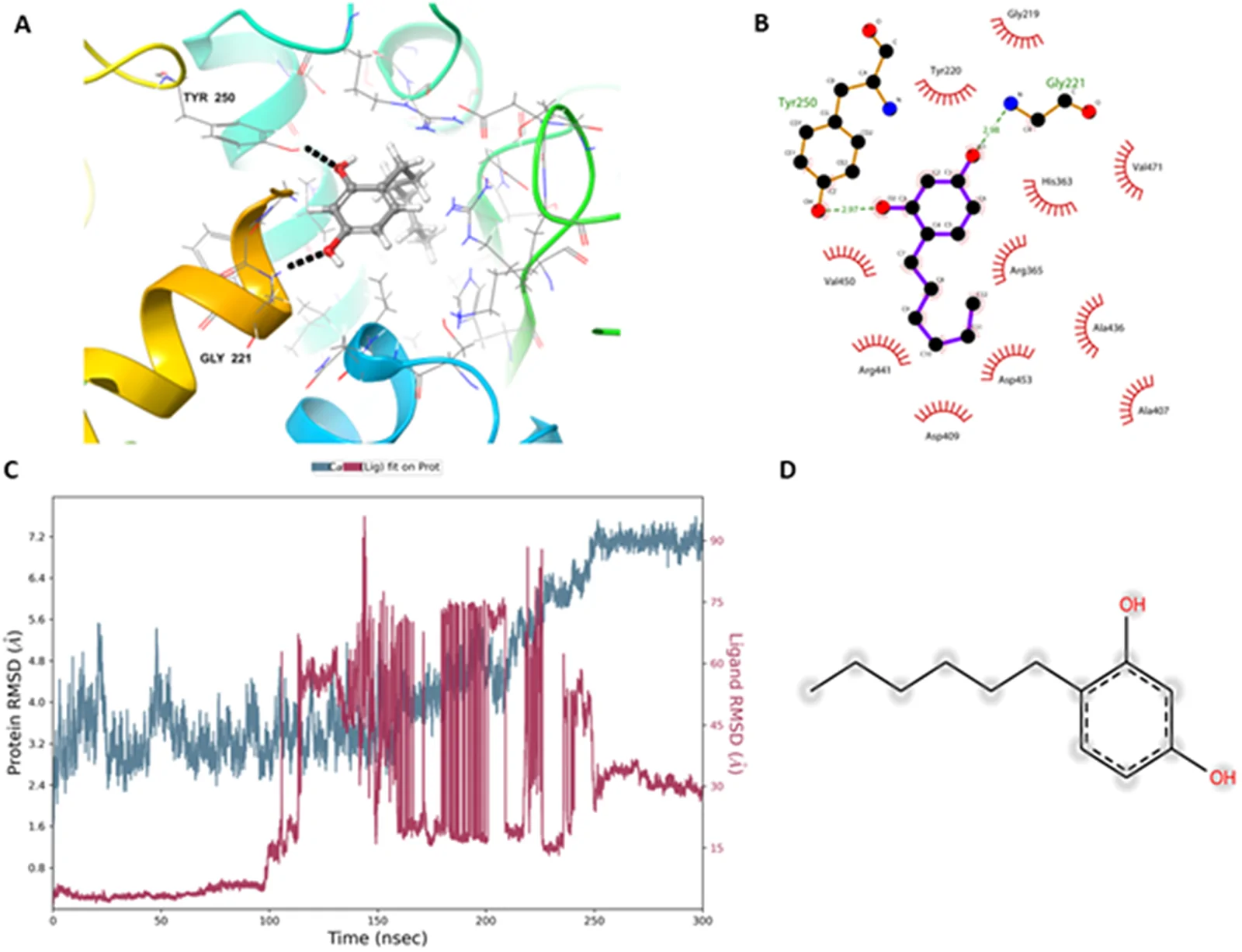
(A) Binding pose of compound 8 in the active site of human FUT8 obtained from molecular docking (black dashed lines indicate hydrogen bonds). (B) Two-dimensional LigPlot interaction map of compound **8**–FUT8 showing hydrogen-bond interactions and bond distances. (C) RMSD profiles of the FUT8 protein backbone (Cα, blue) and compound **8** fitted on FUT8 (red-brown) over the 300 ns molecular dynamics simulation. (D) Two-dimensional summary of protein–ligand interactions observed during the MD trajectory, showing occupancies of more than 30% of the simulation time.

The dynamic stability of the compound **8**–FUT8 complex was evaluated over a 300 ns MD simulation (Fig 20C). During the initial phase of the simulation, the protein RMSD remained relatively stable at approximately 2.5–3.5 Å up to ~150 ns, followed by a gradual increase reaching ~7.0–7.5 Å toward the later stage of the trajectory, indicating late-stage conformational rearrangement of the protein. On the other hand, the ligand RMSD remained low during the early phase but showed pronounced fluctuations after approximately 100 ns, with large excursions observed throughout the middle portion of the simulation. Partial re-stabilization of the ligand RMSD occurred during the final phase; however, significant positional variability persisted, indicating intermittent disengagement and re-association of compound **8** within the binding pocket.

Protein–ligand interaction analysis indicated poor dynamic stability of compound **8** within the FUT8 binding pocket (Fig 20D). Although Gly221 and Tyr250 formed hydrogen bonds in the docking pose, these interactions were not maintained during the MD simulation. Instead, compound **8** displayed mainly transient and water-mediated contacts distributed across multiple residues, with no single interaction showing high occupancy over the trajectory. The total number of protein–ligand contacts fluctuated significantly throughout the simulation, consistent with the pronounced ligand RMSD excursions and intermittent disengagement from the binding site. Quantitative contact frequencies and time-resolved interaction profiles are provided in the Supplementary Information (Figs S31 and S32 in S1 File).

Post-MD MM/GBSA analysis of 201 trajectory frames yielded an average binding free energy of −21.62 ± 12.35 kcal/mol, with values ranging from −44.56 to 0.67 kcal/mol, reflecting significant energetic variability during the simulation. The corresponding no-strain binding free energy was −23.17 ± 13.06 kcal/mol (range: −48.08 to 0.67 kcal/mol). Hydrogen-bond analysis revealed fluctuating interaction numbers throughout the trajectory, with predominantly zero to one hydrogen bond maintained at any given time and only occasional transient increases (Fig S33 in S1 File). Collectively, these results indicate weak and dynamically unstable engagement of compound **8** within the FUT8 active site, characterized by limited persistence of polar contacts, pronounced ligand mobility, and unfavorable binding energetics.

Compounds **1**, **2**, **3**, **4**, **6**, and **7** were able to occupy the donor-binding site of FUT8 and interact with key residues of the enzyme. Furthermore, the closely aligned RMSD values of the protein and the ligand underscore substantial ligand/pocket compatibility, reaffirming the stability of the complex and the convergence of the MD simulation.

### Anti-migratory effects in prostate cancer (PC3) cell line

FUT8 has been reported to be pivotal in cell migration [40], indicating a potential impact on prostate cancer progression. Based on these studies, we evaluated the anti-migratory potential of lead compounds at their IC_50_ concentrations in PC-3 cells using the scratch cell assay. As compounds **2** and **4** were insoluble and their STD-NMR analysis could not be performed, and compounds **5** and **8** were unstable and diffused away from the protein during MD, compounds **1**, **3**, **6**, and **7** were further processed for scratch cell anti-migratory assay. Two controls were used: the negative control (only cells) and the vehicle control (1% DMSO-treated cells), which showed scratch closures of 48.48% and 45.45% after 24 hours and 100% after 48 hours, respectively, validating the migratory capacity of PC3 cells. The scratch closure in cells treated with the compound **7** was 1.75 and 10.37% after 24 and 48 hours, respectively. Compound **3** demonstrated a scratch closure of 3.14 and 7.49% after 24 and 48 hours, respectively, indicating a significant anti-migratory effect. Compounds **1** and **6** demonstrated scratch closure of 5.14% and 7.60% after 24 hours, and 14.18% and 11.47% after 48 hours of treatment, respectively (Fig 21). These results suggested that the anti-migratory effect of these compounds may be due to FUT8 inhibition in PC3 cells. However, further studies are needed to understand the molecular mechanisms underlying this phenomenon.

**Fig 21:**
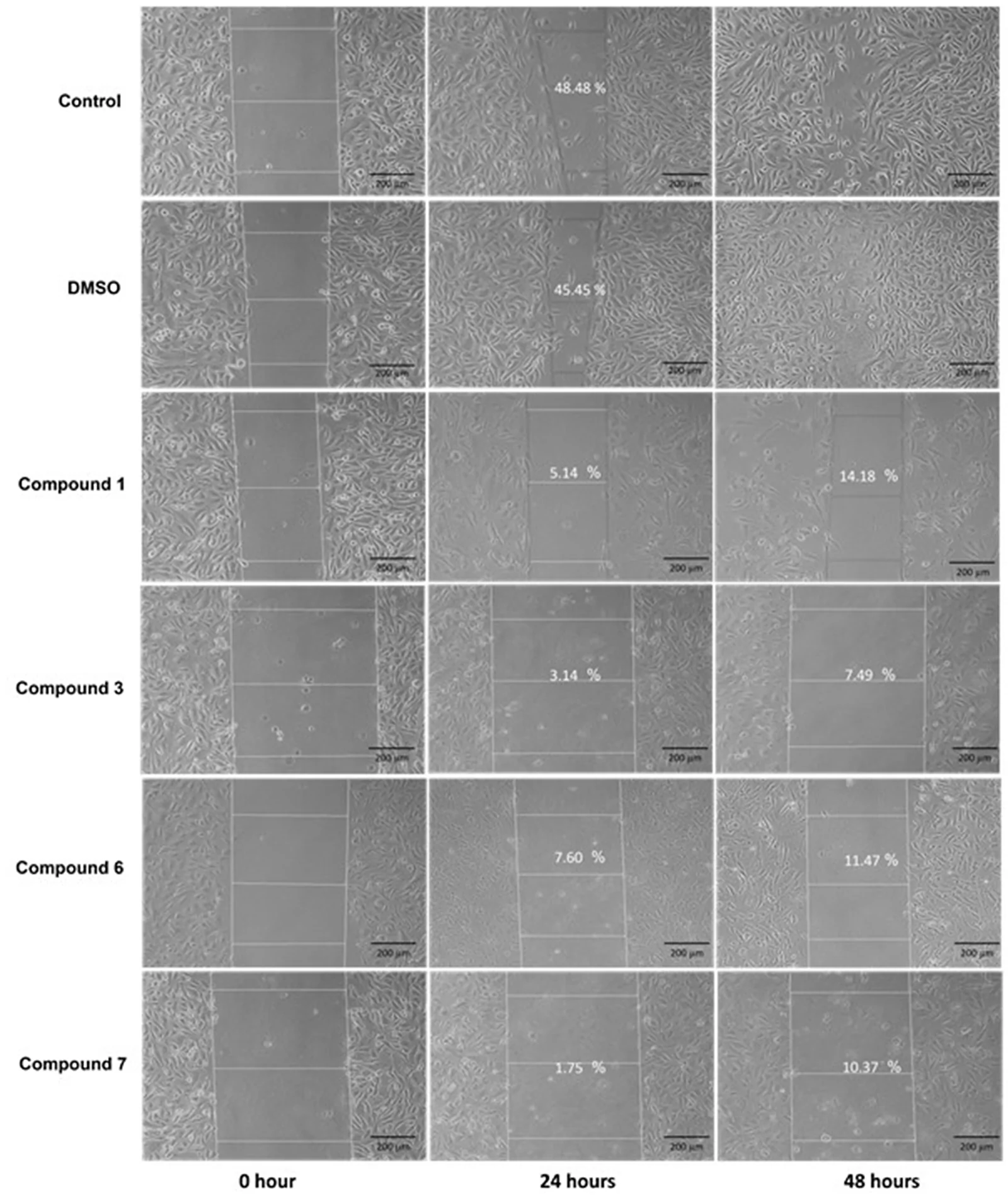
Analysis of the anti-migratory ability of lead compounds by scratch cell assay. Scale bar = 200 μm.

### Correlation between STD-NMR and MD simulation

The complementary use of STD-NMR spectroscopy and molecular docking–molecular dynamics (MD) simulations provided a coherent and mutually supportive interpretation of ligand–FUT8 interactions. Following cell-based and thermal shift assays, STD-NMR analysis of six buffer-soluble compounds (**1**, **3**, **5**, **6**, **7**, and **8**) confirmed direct ligand–FUT8 interactions, whereas compounds **2** and **4** could not be evaluated due to solubility limitations in Tris buffer. While STD-NMR spectroscopy effectively showed binding interactions, it could not resolve residue-specific interactions or active-site involvement, nor detect exchangeable protons such as –OH and –NH_2_. To address these limitations, molecular docking and subsequent MD simulations were employed. Docking results indicated favorable binding poses for all compounds; however, MD simulations further refined these findings, revealing differential binding stability. Compounds **5** and **8** exhibited unstable binding and diffused from the FUT8 binding site during the simulation, whereas compounds **1**, **3**, **6**, and **7** remained stably associated with FUT8 throughout the 300 ns trajectory. Furthermore, the interacting protons of these four compounds (**1**, **3**, **6**, and **7**) with FUT8 in STD-NMR corresponded to hydrophobic regions that maintained persistent contacts during MD simulations, demonstrating strong agreement between experimental and computational analysis. These integrated analyses strengthen the study that compounds **1**, **3**, **6**, and **7** represent the most promising lead candidates.

### Strengths and limitations of the study

A major strength of this study lies in the integrated, multi-assay approach that combines cell-based assays, thermal shift analysis, STD-NMR spectroscopy, molecular docking, and molecular dynamics simulations, thereby enabling cross-validation of ligand–protein interactions from both experimental and computational perspectives. STD-NMR spectroscopy provided direct experimental evidence of binding, while MD simulations enabled residue-level and temporal evaluation of ligand stability within the FUT8 binding site, overcoming key experimental limitations. However, certain limitations remained. STD-NMR spectroscopy is inherently limited by the solubility of compounds in aqueous buffers, the inability to detect exchangeable protons, and the lack of site-specific interaction information. Similarly, molecular docking and MD simulations rely on force-field accuracy and simulation timescales, which may not fully capture long-term conformational dynamics. Despite these limitations, the convergence of STD-NMR interaction patterns with MD-derived stability profiles significantly enhances confidence in the identified lead compounds. Overall, these complementary strategies provide a robust framework for lead validation and rational optimization in FUT8-targeted drug discovery.

## Conclusion

A stepwise screening strategy integrating cellular, biophysical, and computational approaches was applied to identify novel FUT8-targeting anti-cancer candidates. Screening of 265 compounds against PC3 cells identified eight compounds with significant anti-proliferative activity and minimal cytotoxicity toward normal BJ cells. Biophysical characterization using DSF and STD-NMR spectroscopy confirmed direct ligand-FUT8 interactions, with compounds **1**, **3**, **6**, and **7** showing strong saturation transfer effects and inducing thermal conformational destabilization of FUT8. Molecular docking and molecular dynamics simulations further demonstrated stable binding of these compounds within the FUT8 binding site, consistent with experimental interaction patterns. In cell-based assays, these lead compounds also inhibited cancer cell migration. Overall, the convergence of cellular efficacy, biophysical validation, and computational stability supports compounds **1**, **3**, **6**, and **7** as promising FUT8-targeting lead candidates for further optimization and *in vivo* investigation.

## Supporting information

S1 File Supporting Figures S1–S33, Tables S1–S3. (DOCX)
