# Supplementary material for: Identification of new fucosyltransferase-8 inhibitors: An integrated biochemical, biophysical, and computational approach
Source: PLoS One. 2026 Oct 5;21(10):e0349302. doi: 10.1371/journal.pone.0349302 (PMC13637930; doi:10.1371/journal.pone.0349302)
Supplement: Supplementary file 1 [file pone.0349302.s001.docx]

**Identification of New Fucosyltransferase-8 Inhibitors: An Integrated Biochemical, Biophysical, and Computational Approach**

**Supplementary Figures**

**Figure S1.** Supremposed structures of cocrystalized GDP (green color) and docked GDP (Grey) with FUT8.

**Figure S2.** Anti-proliferative effect of compounds on PC-3 cell line. The data are presented as mean±SEM. Data were analyzed using one-way ANOVA followed by Dunnett's test, *P <* 0.05. Compounds vs. 2FF (standard), ****P* < 0.0001, n = 3

**Figure S3.** Representative dose-response curves of compounds with anti-proliferative effects on the PC-3 cell line.

**Figure S4.** Interactions fraction histogram for GDP during the 300 ns molecular dynamics simulation with FUT8, showing residue-wise contacts formed during the trajectory.

**Figure S5.** Time-resolved protein–ligand contact timeline for GDP during the 300 ns molecular dynamics simulation with FUT8, showing total contacts (top) and residue-wise contact occurrences (bottom) across the trajectory.

**Figure S6.** Time-dependent hydrogen-bond numbers between GDP and FUT8 during the 300 ns molecular dynamics simulation.

**Figure S7.** Interactions fraction histogram for 2FF during the 300 ns molecular dynamics simulation with FUT8, showing residue-wise contacts formed during the trajectory.

**Figure S8.** Time-resolved protein–ligand contact timeline for 2FF during the 300 ns molecular dynamics simulation with FUT8, showing total contacts (top) and residue-wise contact occurrences (bottom) across the trajectory.

**Figure S9.** Time-dependent hydrogen-bond numbers between 2FF and FUT8 during the 300 ns molecular dynamics simulation.

**Figure S10.** Interactions fraction histogram for Compound **1** during the 300 ns molecular dynamics simulation with FUT8, showing residue-wise contacts formed during the trajectory.

**Figure S11.** Time-resolved protein–ligand contact timeline for Compound **1** during the 300 ns molecular dynamics simulation with FUT8, showing total contacts (top) and residue-wise contact occurrences (bottom) across the trajectory.

**Figure S12.** Time-dependent hydrogen-bond numbers between Compound **1** and FUT8 during the 300 ns molecular dynamics simulation.

**Figure S13.** Interactions fraction histogram for Compound **2** during the 300 ns molecular dynamics simulation with FUT8, showing residue-wise contacts formed during the trajectory.

**Figure S14.** Time-resolved protein–ligand contact timeline for Compound **2** during the 300 ns molecular dynamics simulation with FUT8, showing total contacts (top) and residue-wise contact occurrences (bottom) across the trajectory.

**Figure S15.** Time-dependent hydrogen-bond numbers between Compound **2** and FUT8 during the 300 ns molecular dynamics simulation.

**Figure S16.** Interactions fraction histogram for Compound **3** during the 300 ns molecular dynamics simulation with FUT8, showing residue-wise contacts formed during the trajectory.

**Figure S17.** Time-resolved protein–ligand contact timeline for Compound **3** during the 300 ns molecular dynamics simulation with FUT8, showing total contacts (top) and residue-wise contact occurrences (bottom) across the trajectory.

**Figure S18.** Time-dependent hydrogen-bond numbers between Compound **3** and FUT8 during the 300 ns molecular dynamics simulation.

**Figure S19.** Interactions fraction histogram for Compound **4** during the 300 ns molecular dynamics simulation with FUT8, showing residue-wise contacts formed during the trajectory.

**Figure S20.** Time-resolved protein–ligand contact timeline for Compound **4** during the 300 ns molecular dynamics simulation with FUT8, showing total contacts (top) and residue-wise contact occurrences (bottom) across the trajectory.

**Figure S21.** Time-dependent hydrogen-bond numbers between Compound **4** and FUT8 during the 300 ns molecular dynamics simulation.

**Figure S22.** Interactions fraction histogram for Compound **5** during the 300 ns molecular dynamics simulation with FUT8, showing residue-wise contacts formed during the trajectory.

**Figure S23.** Time-resolved protein–ligand contact timeline for Compound **5** during the 300 ns molecular dynamics simulation with FUT8, showing total contacts (top) and residue-wise contact occurrences (bottom) across the trajectory.

**Figure S24.** Time-dependent hydrogen-bond numbers between Compound **5** and FUT8 during the 300 ns molecular dynamics simulation.

**Figure S25.** Interactions fraction histogram for Compound **6** during the 300 ns molecular dynamics simulation with FUT8, showing residue-wise contacts formed during the trajectory.

**Figure S26.** Time-resolved protein–ligand contact timeline for Compound **6** during the 300 ns molecular dynamics simulation with FUT8, showing total contacts (top) and residue-wise contact occurrences (bottom) across the trajectory.

**Figure S27.** Time-dependent hydrogen-bond numbers between Compound **6** and FUT8 during the 300 ns molecular dynamics simulation.

**Figure S28.** Interactions fraction histogram for Compound **7** during the 300 ns molecular dynamics simulation with FUT8, showing residue-wise contacts formed during the trajectory.

**Figure S29.** Time-resolved protein–ligand contact timeline for Compound **7** during the 300 ns molecular dynamics simulation with FUT8, showing total contacts (top) and residue-wise contact occurrences (bottom) across the trajectory.

**Figure S30.** Time-dependent hydrogen-bond numbers between Compound **7** and FUT8 during the 300 ns molecular dynamics simulation.

**Figure S31.** Interactions fraction histogram for Compound **8** during the 300 ns molecular dynamics simulation with FUT8, showing residue-wise contacts formed during the trajectory.

**Figure S32.** Time-resolved protein–ligand contact timeline for Compound **8** during the 300 ns molecular dynamics simulation with FUT8, showing total contacts (top) and residue-wise contact occurrences (bottom) across the trajectory.

**Figure S33.** Time-dependent hydrogen-bond numbers between Compound **8** and FUT8 during the 300 ns molecular dynamics simulation.

**Supplementary Tables**

**Table S1**: Melting temperature (Tm) values for all compounds

| **Code** | **Melting Point (°C)** | | | **SD** |
| --- | --- | --- | --- | --- |
| **FUT8** | 46 | 45 | 47 | ± 1.0 |
| **GDP** | 49.35 | 48.75 | 50 | ± 0.62 |
| **2FF** | 45.6 | 44.6 | 46.7 | ± 1.05 |
| **Compound 1** | 43.5 | 41.55 | 45.4 | ± 1.92 |
| **Compound 2** | 45.75 | 44.6 | 46.9 | ± 1.15 |
| **Compound 3** | 44.55 | 44 | 45.1 | ± 0.55 |
| **Compound 5** | 45 | 47 | 43 | ± 2.0 |
| **Compound 6** | 45.3 | 44 | 46.6 | ± 1.3 |
| **Compound 7** | 39 | 38.9 | 39.1 | ± 0.1 |
| **Compound 8** | 37 | 36.7 | 37.3 | ± 0.3 |

**Table S2:** Chemical shift and STD-amplification factors of compounds **1**, **3**, and **5**-**8**.

| **Proton No.** | **Chemical Shift** | **STD amp Factor** |
| --- | --- | --- |
| **Compound 1** | | |
| H-9 | 6.42 | 125.83 |
| H-1 | 6.97 | 138.08 |
| H-10 | 7.64 | 103.66 |
| H-7 | 7.85 | 132.83 |
| H-12 | 8.10 | 71.99 |
| **Compound 3** | | |
| H-7 | 6.85 | 16.23 |
| H-6 | 7.45 | 15.58 |
| H-4 | 7.55 | 29.51 |
| **Compound 5** | | |
| CH_3_ | 1.95 | 26.33 |
| **Compound 6** | | |
| H-1" | 10.00 | 45.62 |
| H-4 | 7.74 | 133.30 |
| H-6 | 7.46 | 72.29 |
| **Compound 7** | | |
| H3'/H5' | 6.89 | 17.71 |
| H-2'/H-6' | 7.01 | 18.45 |
| H-2/H-6 | 7.05 | 17.91 |
| H-3/H-5 | 7.63 | 17.26 |
| **Compound 8** | | |
| H-3, H-5 | 6.40 | 32.14 |
| H-3"/ H-4"/ H-5" | 1.26 | 7.86 |
| H-6" | 0.81 | 12.68 |

**Table S3:** Docking scores, binding free energies of compounds, and residues of FUT8 involved in hydrogen-bond interactions with ligands, along with their bond lengths.

| **Ligands** | **Docking Score** | **MM-GBSA (kcal/mol)** | **Receptor-interacting residues** | **Distance/Bond length** |
| --- | --- | --- | --- | --- |
| GDP | -10.131 | -64.49 | Gly219 | 2.88 |
|  |  |  | Tyr250 | 2.98 |
|  |  |  | His363 | 3.17 |
|  |  |  | Arg365 | 2.86 |
|  |  |  |  | 2.96 |
|  |  |  | Lys369 | 2.70 |
|  |  |  | Asp453 | 2.80 |
|  |  |  |  | 2.98 |
|  |  |  | Ser469 | 3.11 |
| 2FF | -5.378 | -20.19 | His363 | 2.74 |
|  |  |  | Asp453 | 2.58 |
| Compound **1** | -5.421 | -34.81 | Arg365 | 3.16 |
| Compound **2** | -4.697 | -43.83 | Arg441 | 2.83 |
|  |  |  | Gln470 | 2.95 |
| Compound **3** | -5.940 | -42.52 | Tyr250 | 2.76 |
| Compound **4** | -5.326 | -21.60 | Tyr250 | 2.88 |
|  |  |  | Asp453 | 2.63 |
| Compound **5** | -6.173 | -11.63 | Asp453 | 2.60 |
| Compound **6** | -5.310 | -23.90 | Ser446 | 3.22 |
| Compound **7** | -7.286 | -54.31 | Asp453 | 2.44 |
| Compound **8** | -4.990 | -30.68 | Gly221 | 2.98 |
|  |  |  | Tyr250 | 2.97 |
